# Plasmonically Enhanced Hydrogen Evolution on Anisotropic AuPt Nanowires with Submonolayer Pt Surface Coverage

**DOI:** 10.1002/smll.202510990

**Published:** 2025-10-30

**Authors:** IbrahiM Abdelsalam, Shiqi Wang, Hugo L. S. Santos, Ella Kitching, Pranava Pakala, Mykhailo Chundak, Mikko Ritala, Sarah J. Haigh, Thomas J. A. Slater, Pedro H. C. Camargo

**Affiliations:** ^1^ Department of Chemistry University of Helsinki A.I. Virtasen aukio 1, PO Box 55 Helsinki FIN‐0014 Finland; ^2^ Cardiff Catalysis Institute School of Chemistry Cardiff University Cardiff CF10 3AT UK; ^3^ Department of Materials University of Manchester Manchester M13 9PL UK

**Keywords:** AuPt nanowires, density functional theory, hydrogen evolution reaction, plasmonic catalysis, submonolayer catalysts

## Abstract

The rational design of electrocatalysts that efficiently harness plasmonic excitation for electrocatalytic hydrogen production from water remains challenging. Here, guided by density functional theory (DFT) predictions, anisotropic AuPt nanowires are systematically synthesized with precise monolayer and submonolayer Pt surface coverage on Au nanowire templates. Under visible‐light‐driven plasmonic excitation, these catalysts exhibited high hydrogen evolution reaction (HER) activity, achieving mass activities up to 9.3 A mg^−1^_Pt_ at −0.05 V vs RHE, representing a ≈7‐fold enhancement over commercial Pt/C catalysts and surpassing spherical AuPt nanoparticles. Detailed electron microscopy, spectroscopy, and electrochemical analyses indicated that the submonolayer Pt coverage provided more isolated catalytic sites, optimal electronic coupling, and preserved plasmonic properties. DFT calculations reveal pronounced electronic redistribution at Au–Pt interfaces, raise Pt d‐band centers, and ideal Gibbs free energies for hydrogen adsorption. This synergistic combination of catalytic and plasmonic properties represents a promising strategy to substantially reduce precious metal usage without compromising catalytic performance, offering a robust framework for designing electrocatalysts for renewable energy conversion.

## Graphical abstract

Au nanowires decorated with ultralow, submonolayer Pt coverages are demonstrated as efficient plasmonic antenna–reactor systems for the hydrogen evolution reaction (HER) in acidic media. The architecture preserves Au plasmon resonances while maximizing Pt utilization, leading to light‐enhanced catalytic activity. This study establishes a distinct design principle for efficient hydrogen production at minimal Pt loadings.

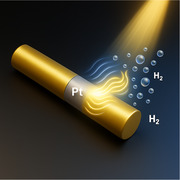

## 1 Introduction

The production of hydrogen through electrocatalytic water splitting, comprising the hydrogen and oxygen evolution reactions (HER and OER, respectively), is a critical pathway to decarbonizing the chemical and energy sectors.^[^ ^1^, ^2^, ^3^ ^]^ Yet, its widespread implementation is constrained by the need for precious‐metal catalysts, such as platinum (Pt) for the HER, which remain costly, scarce, and geographically concentrated.^[^ ^4^, ^5^ ^]^ A central challenge is thus to develop catalyst architectures that dramatically improve Pt utilization while maintaining or enhancing activity and stability.^[^ ^6^, ^7^, ^8^, ^9^ ^]^

Plasmonic electrocatalysis, which harnesses localized surface plasmon resonance (LSPR) in metallic nanostructures to generate energetic charge carriers and localized heating under visible light, offers a compelling strategy to address this limitation.^[^ ^10^, ^11^, ^12^ ^]^ Antenna–reactor designs that integrate a plasmonic metal (e.g., Au or Ag) with catalytically active sites (e.g., Pt) can synergistically enhance reaction kinetics and selectivity, enabling, at least in principle, a reduction in the use of catalytic noble metals.^[^ ^13^, ^14^, ^15^, ^16^ ^]^ Such hybrid nanostructures offer the dual advantages of harvesting optical energy and maximizing catalytic activity.^[^ ^13^, ^14^, ^15^ ^]^ However, realizing and maximizing this potential requires precise control over surface morphology, electronic structure, and metal distribution at the atomic scale: conditions that are difficult to achieve, especially under ultralow catalyst loadings.^[^ ^17^, ^18^, ^19^ ^]^

Recent studies, including our previous work on AuPt nanospheres, have demonstrated that sparse and non‐uniform distributions of Pt on Au cores can significantly enhance HER activity under LSPR excitation, highlighting the importance of nanoscale interface engineering.^[^ ^17^, ^19^, ^20^ ^]^ Yet, these concepts remain largely unexplored in anisotropic nanostructures such as nanowires, which offer unique advantages including enhanced light–matter interaction and facet‐dependent electronic properties.^[^ ^21^, ^22^, ^23^, ^24^ ^]^ Critically, the influence of morphology, particularly anisotropy, on plasmonic coupling and catalytic performance under ultralow Pt loading has yet to be systematically examined. Previous studies of AuPt nanostructures have largely focused on spherical morphologies or higher Pt loadings, where continuous shells dominate surface chemistry and plasmonic responses are dampened. While AuPt nanowires themselves have been reported, the systematic investigation of anisotropic AuPt nanowires with submonolayer Pt surface coverage in plasmon‐enhanced HER remains limited.^[^ ^17^, ^25^, ^26^, ^27^ ^]^

Notably, prior studies have underscored the importance of Au–Pt coordination in nanowire systems. For example, ultrathin Pt–Au alloy nanowires with (111)‐dominant facets have been reported where Pt was the primary catalytic component and Au served as an alloying element, demonstrating enhanced activity for oxygen reduction and alcohol oxidation.^[^ ^17^ ^]^ More recently, ultrathin Au_3_Pt twin nanowires for spontaneous hydrogen production coupled with glucose oxidation have been developed, emphasizing coordination tuning, tensile strain, and Pt‐to‐Au site transfer as mechanisms for improved stability and selectivity.^[^ ^25^ ^]^ In parallel, plasmonic bimetallic nanostructures such as Pd–Au nanorods have been explored for ethanol oxidation under illumination.^[^ ^28^ ^]^ Other studies have also reported Au–Pt nanostructures in different contexts: Au@Pt nanostructures for ethanol oxidation in fuel cells,^[^ ^27^ ^]^ Au–Pt nanotube networks as stretchable biosensing electrodes,^[^ ^29^ ^]^ and synthesized ultrathin Au–Pt nanowires with stepped Pt sites for alcohol electro‐oxidation.^[^ ^26^ ^]^ While these works provide valuable insights into alloying, strain, and hot‐carrier processes, they typically involve continuous or thick Pd/Pt shells, focused on oxidation or sensing reactions, and rely on relatively high precious‐metal contents that dampen plasmonic responses.

In contrast, here we introduce a distinct design paradigm in which anisotropic helical Au nanowire networks serve as plasmonic scaffolds decorated with ultralow, submonolayer Pt coverages. To address this gap, we designed and investigated a series of AuPt nanowires with controlled low Pt contents (<10 at.%). By employing density functional theory (DFT) calculations, we identified optimal atomic‐scale surface compositions expected to maximize catalytic activity and plasmonic enhancement. Guided by these computational insights, we synthesized bimetallic nanowires via a template‐directed co‐reduction strategy, yielding catalysts with well‐defined surface morphology and Pt coverages ranging from ≈1–2 atomic layers down to submonolayer levels. The electrocatalytic performance of the produced nanowires was evaluated under both dark and visible‐light‐driven plasmonic excitation conditions. Our results revealed that the electrocatalytic performance displayed a strong dependence on Pt loading, surface distribution, and morphology, as predicted by our DFT calculations. Specifically, the AuPt nanowires with submonolayer Pt coverage exhibited superior HER performance, achieving overpotentials as low as 35 mV at −10 mA cm^−2^, compared to 66 mV for equivalent spherical AuPt nanoparticles and closely matching the 28 mV of commercial Pt/C catalysts. Under visible‐light illumination, the nanowires demonstrated a pronounced plasmonic enhancement, achieving a mass activity of 9.3 A mg^−1^_Pt_ at −0.05 V vs RHE, ≈7‐fold higher than commercial Pt/C catalysts under identical conditions (the nanospheres showed negligible response to illumination). This study represents an advancement within the established field of plasmonic electrocatalysis rather than a conceptual breakthrough. It builds upon recent progress in understanding energy and charge redistribution in multimetallic plasmonic systems^[^ ^30^, ^31^, ^32^, ^33^, ^34^ ^]^ and integrates anisotropic Au nanowire scaffolds with discontinuous submonolayer Pt decoration to couple efficient plasmonic excitation with minimized Pt utilization for hydrogen evolution in acidic media. Our findings not only elucidate fundamental structure–activity–plasmonic relationships but also establish broadly applicable design principles for developing efficient, cost‐effective catalysts to sustainably meet future energy demands.

## 2 Results and Discussion

**Figure 1:**
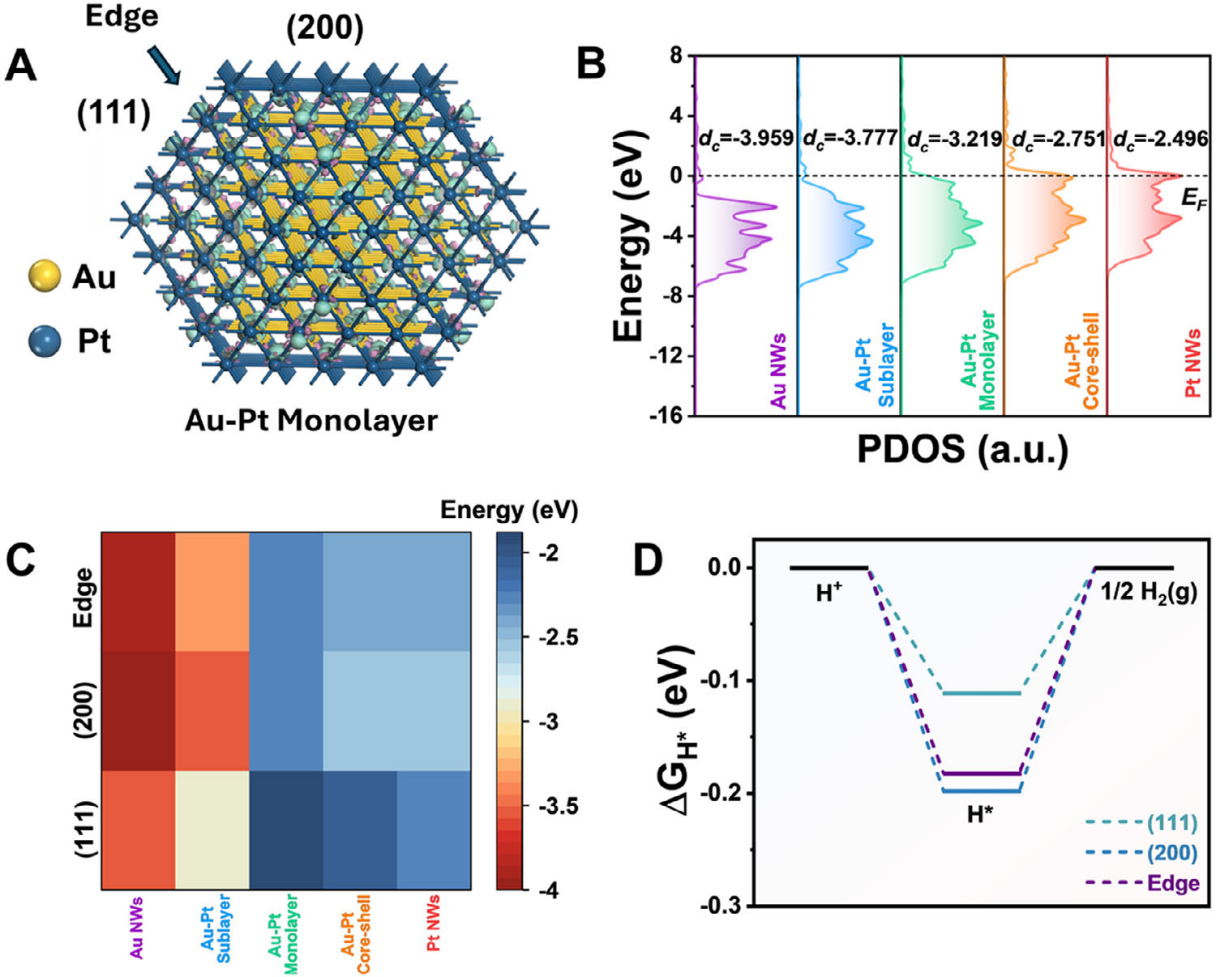
DFT‐based analysis of surface configurations and HER activity in AuPt nanowires. A) Atomic models and corresponding charge density difference (CDD) maps for AuPt nanowires with a monolayer configuration. Exposed facets include (111) and (200) surfaces. The wire is viewed along the longitudinal axis. Green and pink isosurfaces indicate regions of electron accumulation and depletion, respectively (isosurface value = 0.05 e Å^−3^). B) Projected density of states of the d‐orbitals (PDOS) highlighting the evolution of electronic structure across different configurations. C) Calculated d‐band center values for various catalytic sites on (111), (200), and edge positions on Au nanowires, Au‐Pt sublayer, Au‐Pt monolayer, and Au‐Pt core‐shell models, and Pt nanowires. This illustrates the sensitivity of surface coordination to Pt coverage. D) Gibbs free energy profiles (ΔG_H*_) for hydrogen adsorption at Pt sites in the Au–Pt monolayer model, showing facet‐dependent catalytic behavior relevant to HER activity.

The rational design of electrocatalysts that efficiently couple plasmonic excitation with catalytic function requires an understanding of how surface morphology and distribution influence catalytic activity at the atomic level.^[^ ^14^ ^]^ Building upon the insights from our previous studies of spherical AuPt nanoparticles,^[^ ^19^ ^]^ we employed DFT simulations to identify optimal atomic configurations and Pt surface coverages for the AuPt nanowires. Theoretical models comprising two main exposed surface facets – (111) and (200) – alongside corresponding edge sites were constructed (Figure S1A, Supporting Information; **Figure** **1**A). These models span various degrees of Pt loadings and coverage, and were denoted as Au nanowires (control sample, no Pt), Au‐Pt Sublayer (submonolayer coverage), Au‐Pt Monolayer (monolayer coverage), Au‐Pt Core‐shell (multiple layer coverage), and Pt nanowires (control sample, pure Pt).

Analysis of charge density difference (CDD) diagrams revealed significant electronic redistribution at the Au‐Pt interfaces in the Au‐Pt Sublayer, Au‐Pt Monolayer, and Au‐Pt. Core‐shell configurations, contrasting markedly with monometallic Au and Pt nanowires^[^ ^35^ ^]^ (Figure 1A). The pronounced regions of electron accumulation and depletion indicate substantial electron density transfer from Au cores toward Pt surface sites. Furthermore, electron localization function (ELF) analysis revealed more delocalized electrons around surface Pt atoms, particularly at the (111) sites (Figure S1B, Supporting Information).^[^ ^36^ ^]^ These electronic redistributions suggest enhanced LSPR‐induced hot electron mobility toward Pt catalytic sites under visible light excitation, potentially leading to accelerated reaction kinetics.

To further explore the electronic properties of the various AuPt NW models, we analyzed the projected partial density of states (PDOS) of the d‐orbitals and corresponding d‐band centers (d*_c,_* Figure 1B). Compared to pure Au nanowires, all AuPt models exhibited an elevated d‐band center, that increased with the Pt content, indicative of enhanced adsorption affinity toward critical reaction intermediates.^[^ ^37^ ^]^ Analysis of PDOS and d‐band centers across distinct surface configurations, including (111), (200), and edge positions, underscored substantial variability dependent upon facet orientation (Figure 1C; Figure S2, Supporting Information). Notably, (111) facets consistently demonstrated higher d‐band centers compared to (200) facets and edge sites, with the (111) facets in Au‐Pt Monolayer configuration exhibiting the highest d‐band center and thus potential for superior catalytic activity.

Given these findings, the Au‐Pt Monolayer model was selected for a more detailed analysis of catalytic activity. Calculations of the Gibbs free energies for hydrogen adsorption (ΔG_H*_) clearly indicated that Pt sites on (111) facets (−0.111 eV) exhibited notably lower ΔG_H*_ values compared to sites on (200) facets (−0.198 eV) and edge positions (−0.182 eV) (Figure 1D). This suggests that the (111) facet Pt sites can contribute more strongly to the enhanced HER performance.^[^ ^38^ ^]^ Consequently, our DFT results indicate that the Pt composition and crystal structure at the surface are expected to impact HER activity, with Pt monolayer coverage on exposed (111) facets being the most favorable configuration for high catalytic activity.

Following the insights provided by our computational results, we proceeded to experimentally synthesize bimetallic AuPt nanowires with controlled Pt surface coverage. Utilizing a co‐reduction method that exploits the difference in reduction potentials between Au and Pt precursors, we synthesized AuPt nanowires with ultralow Pt loadings (the Pt atomic % was varied from 8 to 1). In this synthesis, α‐naphthol acted both as a shape‐directing agent and a reducing agent at 70 °C over a period of 12 h,^[^ ^20^ ^]^ enabling controlled growth of Au nanowires with varying surface compositions of Pt. Specifically, we prepared three distinct samples with controlled Au:Pt ratios, designated as Au_92_Pt_8_, Au_98.5_Pt_1.5_, and Au_99_Pt_1,_ based on the elemental atomic percentages as determined by microwave plasma atomic emission spectroscopy (MP‐AES). A control sample comprised of pure Au nanowires was also prepared. Powder X‐ray Diffraction (XRD) data showed that only diffraction peaks assigned to face centred cubic (*fcc*) Au from all NW compositions due to the low Pt loading in these samples (Figure S3, Supporting Information). Moreover, all the AuPt nanowires, as well as the control Au nanowires sample, displayed similar morphological features (Figure S4, Supporting Information), characterized by an undulating nanowire morphology with widths ranging from 5 to 7 nm (Figure S5, Supporting Information) and lengths extending to several tens of nanometers.

**Figure 2:**
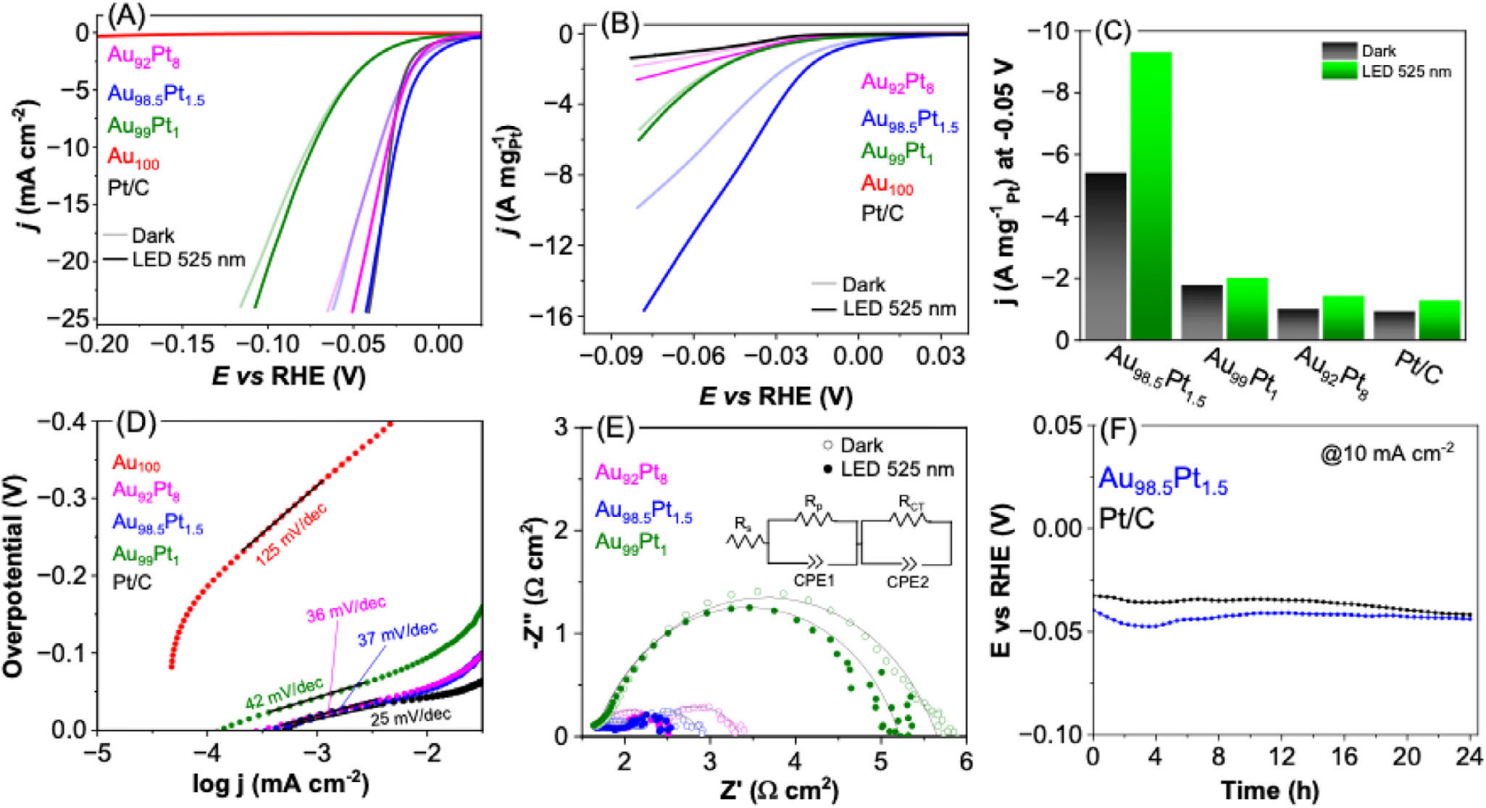
Electrocatalytic performance of AuPt nanowires with varying Pt coverage for the HER. A) LSV curves normalized by geometric electrode area. B) LSV curves normalized by Pt mass, illustrating intrinsic activity. C) mass activity at −0.05 V_RHE_. D) Tafel plots derived from area‐normalized LSVs, highlighting differences in reaction kinetics across catalysts. E) Nyquist plots from EIS, revealing charge transfer resistances. F) Chronoamperometry curves at −10 mV vs RHE, assessing catalytic stability. Measurements were performed in Ar‐saturated 0.5 m H_2_SO_4_ using Pt/C, Au_92_Pt_8_, Au_98.5_Pt_1.5_, Au_99_Pt_1_, and Au_100_ (pure Au) NWs.

We next evaluated the electrocatalytic performance of AuPt nanowires toward the HER as a function of Pt content, under both dark conditions and visible‐light plasmonic excitation (525 nm, 68 mW cm^−2^). The results were benchmarked against a commercial Pt/C catalyst (20 wt.% Pt). Linear sweep voltammetry (LSV) curves normalized by geometric electrode area (**Figure** **2**A) showed that under dark conditions, both Au_92_Pt_8_ and Au_98.5_Pt_1.5_ nanowires delivered HER activities comparable to Pt/C, with overpotentials of 35 and 36 mV at −10 mA cm^−2^, respectively, vs 28 mV for Pt/C. However, when normalized by Pt mass (Figure 2B), significant differences emerged, with Au_98.5_Pt_1.5_ nanowires achieving the highest enhancement in mass activity over Pt/C, outperforming both Au_92_Pt_8_ and Au_99_Pt_1_. These results highlight that controlling the Pt composition enables the optimization of HER performance, with performance reaching a maximum where the Au nanowires contain 1.5 at% Pt. The intrinsic activity of these catalysts was further quantified by evaluating the mass activity at −0.05 V_RHE_ (Figure 2C). Under dark conditions, Au_98.5_Pt_1.5_ NWs exhibited a mass activity of −5.42 A mg^−1^Pt, substantially higher than both Pt/C (−0.94 A mg^−1^Pt) and the other AuPt nanowire compositions (−1.01 and −1.78 A mg^−1^Pt for Au_92_Pt_8_ and Au_99_Pt_1_, respectively). It is important to clarify that mass activities were normalized to Pt content, consistent with standard practice for benchmarking HER electrocatalysts, since Pt is the catalytically active species (Au has no activity under the employed conditions as seen in Figure 2A). This normalization highlights how efficiently each Pt atom is utilized, which is the key metric for evaluating low‐Pt designs.

A wavelength of 525 nm was selected for photocatalytic investigations based on the transverse LSPR band of the nanowires observed in UV–vis spectra (Figure S6, Supporting Information).^[^ ^39^, ^40^ ^]^ Under illumination, all AuPt nanowires displayed enhanced HER activity relative to dark conditions, with the most pronounced enhancement observed for the Au_98.5_Pt_1.5_ nanowires (Figure 2B,C). The mass activity of Au_98.5_Pt_1.5_ nanowires increased to 9.3 A mg^−1^_Pt_, representing a ≈1.7‐fold enhancement relative to dark conditions and a 7.2‐fold improvement over Pt/C under equivalent light exposure. While the absolute differences in overpotentials between dark and illuminated conditions appear modest in geometric area‐normalized plots (Figure 2A), normalization by Pt mass reveals statistically significant light‐induced enhancements, particularly for the Au_98.5_Pt_1.5_ nanowires (1.7‐fold relative to dark, 7.2‐fold relative to Pt/C, Figure 2C). These results indicate that the observed effect is not an artifact of scaling but a genuine plasmon‐mediated enhancement. We note that both hot‐carrier activation and local photothermal heating can contribute under LSPR excitation, and fully disentangling their roles remains highly challenging.^[^ ^41^, ^42^, ^43^ ^]^ Nevertheless, our DFT simulations and spectroscopic data confirm charge redistribution at Au–Pt interfaces and the preservation of Au LSPR features, supporting hot‐carrier transfer as a plausible contributor to the enhanced activity in the submonolayer Pt regime together with photothermal heating.

While normalization to Pt mass is the accepted standard for benchmarking HER electrocatalysts, since Pt constitutes the active catalytic component, it is important to acknowledge that Au is itself an expensive noble metal. Therefore, the present AuPt nanowire catalysts are not proposed as immediately viable for large‐scale deployment. Instead, our study should be viewed as a model platform that establishes a general design principle: dispersing submonolayer Pt onto a plasmonic scaffold maximizes catalytic activity while preserving optical enhancement. The concept is transferable in principle to alternative, more earth‐abundant plasmonic materials such as Ag, Cu, or Al, which could reduce costs while maintaining the essential antenna–reactor synergy. Thus, the key contribution of this work lies not in demonstrating immediate economic competitiveness, but in providing atomic‐level mechanistic insight and structural guidelines for the rational development of low‐Pt plasmonic electrocatalysts.

To contextualize the performance of the Au_98.5_Pt_1.5_ NWs, we compared our results with representative HER electrocatalysts reported in recent literature (Table S2, Supporting Information), showing that Au_98.5_Pt_1.5_ NWs deliver overpotentials and mass activities that are competitive with state‐of‐the‐art catalysts with higher Pt loadings.

Further insights into the electrocatalytic mechanism were obtained from Tafel analysis (Figure 2D). The Tafel slopes, calculated from LSV curves normalized by geometric area, indicated rapid kinetics for Au_98.5_Pt_1.5_ and Au_92_Pt_8_ nanowires, similar to that of the Pt/C catalyst. Electrochemical impedance spectroscopy (EIS) measurements (Figure 2E) revealed lower charge transfer resistances for Au_98.5_Pt_1.5_ and Au_92_Pt_8_nanowires, respectively, indicating efficient electron transfer, thus facilitating faster HER kinetics. The fitted EIS parameters are summarized in Table S3 (Supporting Information). In all cases, the charge‐transfer resistance (R_ct_) decreased under 525 nm LED illumination compared to the dark, indicating improved interfacial charge transport upon plasmon excitation. For example, R_ct_ values decreased from 4.02 → 3.52 Ω·cm^2^ (Au_99_Pt_1_), 0.74 → 0.53 Ω·cm^2^ (Au_98.5_Pt_1.5_), and 0.99 → 0.54 Ω·cm^2^ (Au_92_Pt_8_). The strongest effect was observed for Au98.5Pt1.5, consistent with its superior light‐enhanced HER activity. These results confirm that plasmonic excitation contributes to facilitating charge transfer at the AuPt/electrolyte interface

As the most active catalyst in our series, the stability of the Au_98.5_Pt_1.5_ nanowires was assessed via chronoamperometry at a fixed current density of −10 mA cm^−2^ over a 24‐h period under dark conditions and benchmarked against commercial Pt/C (Figure 2F). We deliberately performed long‐term measurements in the absence of illumination to provide a conservative benchmark and to decouple electrochemical durability from transient photothermal or irradiation‐induced fluctuations, which can complicate stability interpretation. The AuPt nanowires exhibited stable catalytic activity throughout the test and showed comparable performance to Pt/C. HRTEM analyses after the durability test (Figure S7, Supporting Information) confirmed the structural preservation of the nanowires, with no observable surface etching, aggregation, or fragmentation.

We note that Faradaic efficiency (FE) and gas chromatography (GC) measurements of hydrogen production were not performed in this study. However, under the acidic conditions employed (and with Pt‐based catalysts, the HER is well established to proceed with ≈100% FE.^[^ ^44^, ^45^, ^46^ ^]^ The measured overpotentials, Tafel slopes, and stability over 24 h are fully consistent with HER being the dominant process. While direct quantification would further validate this, we consider the risk of significant side reactions to be negligible under these conditions. We therefore interpret the observed currents as corresponding to HER.

**Figure 3:**
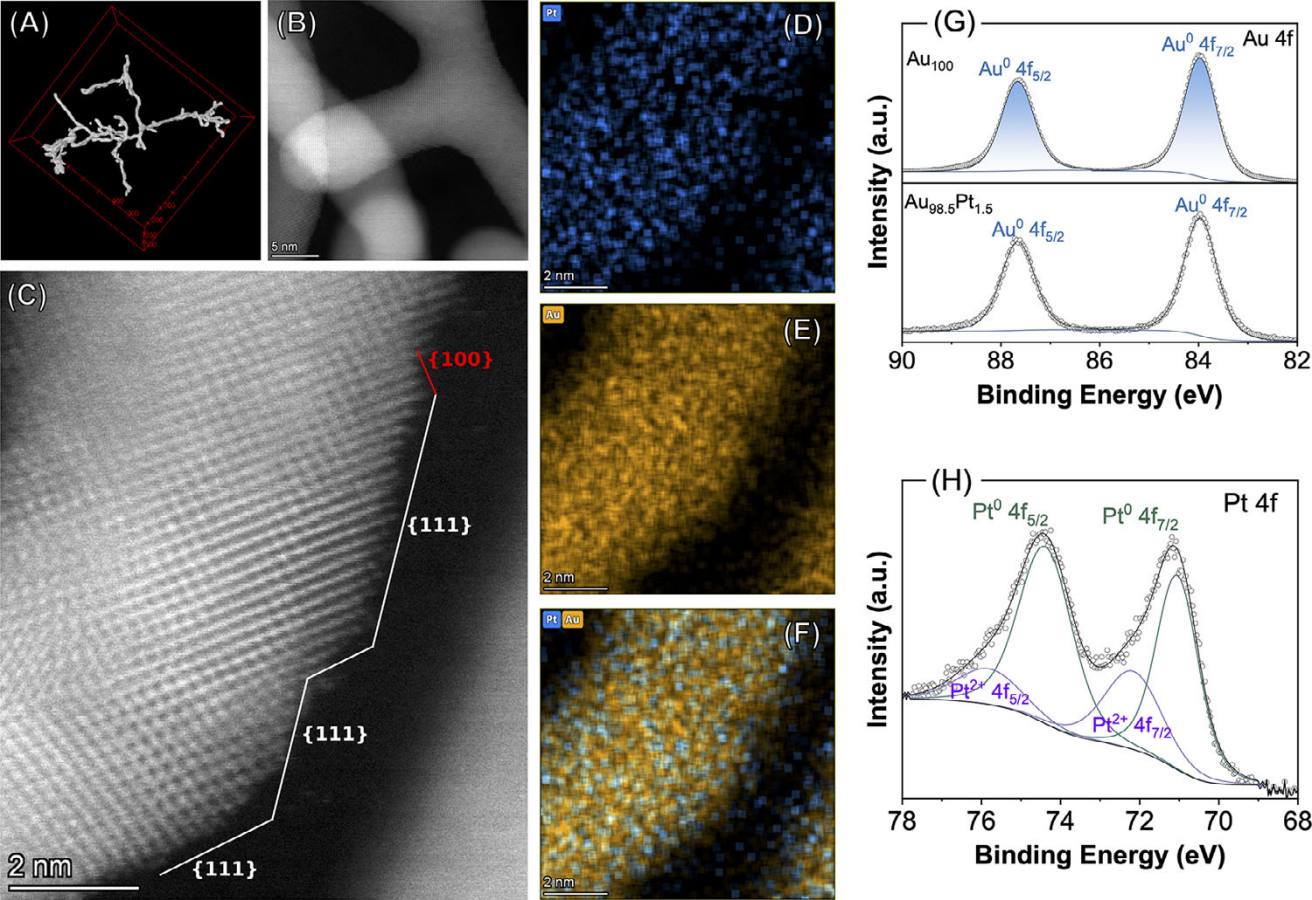
Structural and compositional analysis of Au_98.5_Pt_1.5_ NWs. A) Tomographic render STEM‐HAADF image revealing the uniform nanowire thickness and undulating interconnected morphology (see Movie S1). B) HAADF‐STEM image of a representative nanowire segment. C) Atomic‐resolution HAADF‐STEM image showing the dominance of {111} surface facets, with occasional presence of {100} planes. D–F) STEM‐EDS elemental maps showing the distribution of (D) Pt, (E) Au, and (F) the Au–Pt overlay. The results confirm that there is no continuous coverage of Pt, indicating a dispersed submonolayer configuration, consistent with a calculated Pt surface coverage below 20 nm. G,H) High‐resolution XPS spectra of (G) Au 4f and (H) Pt 4f core levels. In (G), spectra for monometallic Au nanowires are included for comparison. The Pt 4f spectrum in (H) indicates the presence of both metallic Pt⁰ and oxidized Pt^2^⁺ species, consistent with high surface exposure and submonolayer distribution.

To better understand the structural origins of the higher catalytic performance observed for Au_98.5_Pt_1.5_ relative to the other samples, we undertook a comprehensive structural analysis combining 2D and 3D imaging techniques. TEM and HAADF‐STEM imaging (**Figure** **3**A–C; Figure S8, Supporting Information) revealed that the Au_98.5_Pt_1.5_ nanowires possess an undulating morphology, with the wires having a narrow width distribution and an atomically coherent polycrystalline *fcc* structure, containing frequent twins and stacking faults. Frequent twinning within the nanowire‘s *fcc* atomic crystal structure enables a high proportion of the most stable {111} surface facets despite the irregular morphology. Atomic‐resolution imaging confirmed the predominance of exposed {111} surface facets, with minor contributions from {100} planes (Figure 3C; Figure S8, Supporting Information) with d‐spacings of ≈0.24 and ≈0.14 nm, corresponding to {111} and {200} planes, respectively, of *fcc* Au. Importantly, the facet distribution aligns with the DFT predictions that {111}‐terminated Pt sites provide optimal HER activity.

To gain deeper insight into the 3D architecture, the continuity of the helical nanowire networks, and their implications for electrocatalytic performance, we performed electron tomography using HAADF‐STEM (Figure S9, Supporting Information). The 3D imaging revealed an entangled, yet spatially open network of interconnected wires. This geometry enables high surface accessibility and efficient electrolyte penetration while minimizing diffusion limitations.^[^ ^17^, ^25^, ^26^ ^]^ The 3D analysis also confirmed that the individual nanowires maintain consistent diameters and curvature throughout their volume, corroborating the structural coherence observed in 2D projections. These interconnected undulating wires contribute to enhanced charge transport pathways and mechanical resilience during operation, offering a structural rationale for the high activity and stability of the catalysts.

We note that electrochemical surface area (ECSA) determination via hydrogen underpotential deposition is not reliable for these nanostructures, since the Pt coverage is discontinuous and too dilute to yield well‐defined Hupd features, while Au does not provide a clear hydrogen adsorption signal. Similarly, turnover frequency (TOF) values would require precise quantification of the number of accessible Pt sites, which cannot be robustly defined under submonolayer decoration. For this reason, we report Pt mass‐normalized activity as the most rigorous and widely adopted benchmarking metric for low‐Pt HER catalysts. The structural advantages of the 3D nanowire networks, including high accessibility and porosity, are instead supported by direct morphological evidence from SEM, TEM, and tomography.

Further, STEM‐energy‐dispersive X‐ray spectroscopy (STEM‐EDS) mapping illustrates that, while Au was distributed homogeneously across the nanowires (Figure 3E), X‐ray counts of Pt were too low to conclusively locate Pt atoms (Figure 3D), with no evidence indicating the presence of continuous Pt layers. In addition, atomic resolution STEM imaging revealed isolated atomic species that were mobile on the nanowire surfaces under the electron beam (Figure S10, Supporting Information), suggesting that Pt may be present as isolated single atoms on the nanowire surfaces. However, the similar atomic number of Pt and Au makes it impossible to unambiguously assign the individual surface atoms to Pt or Au from STEM‐HAADF imaging. Isolated single atoms were not found on Au nanowires (Figure S11, Supporting Information), suggesting that the isolated atoms observed are likely to be Pt. To avoid overinterpretation, and in the absence of atomic‐resolution spectroscopic techniques such as EXAFS, we conservatively refer to these surface features as submonolayer distributions. Nonetheless, the more discrete and sparse Pt distribution suggests effective formation of more isolated catalytic sites relative to a monolayer or core‐shell coverages, essential for efficient plasmonic enhancement and catalytic activity.^[^ ^47^, ^48^ ^]^ Equivalent 2D and 3D imaging of the monometallic Au nanowires used as control samples and 2D imaging of the other Au‐Pt composition (Figures S11–S14, Supporting Information) revealed all samples had similar structure and morphological features, individual nanowires have a polycrystalline *fcc* atomic structure dominated by {111} surface facets, the nanowires have consistent size distributions and spiral in an irregular manner to form an interconnected network. This gives confidence that the change in catalytic performance is a result of the surface Pt content, not a result of a change in the native Au nanowire morphology.

Although direct visualization of 1–2 atomic layer Pt distributions is inherently challenging, multiple complementary observations support submonolayer Pt decoration rather than continuous coverage at the surface. STEM‐EDS maps show Pt signals localized at the surface, HAADF‐STEM images reveal faint contrast features corresponding to isolated surface atoms or small clusters, and XPS spectra indicate characteristic binding energy shifts consistent with submonolayer Pt. Moreover, the preservation of strong Au plasmon resonances in the UV–vis spectra would not be expected if a continuous Pt shell were present.

The X‐ray photoelectron spectroscopy (XPS) Au 4f spectrum of Au_98.5_Pt_1.5_ (Figure 3G, bottom trace) displayed well‐defined doublets assigned to Au 4f_7/2_ and 4f_5/2_ characteristic of metallic Au^0^. No significant shifts were detected relative to the pure Au nanowires (Figure 3G, top trace). The Pt 4f region (Figure 3H) exhibited main signals at 71.06 and 74.42 eV that can be assigned to the Pt 4f_7/2_ and 4f_5/2_ doublets of Pt. Also, a lower intensity doublet at 72.23 and 75.93 eV that can be assigned to oxidized Pt^2+^ species was detected. The presence of Pt^2+^ suggests partial surface oxidation that can be attributed to the ultralow Pt content, in which the dilute distribution of Pt on Au can favor the formation of Pt^2+^ and sites that are more susceptible to the formation of PtO and Pt(OH)_2_ species.^[^ ^19^, ^48^ ^]^ The detection of both Pt^0^ and Pt^2+^ further supports the interpretation from STEM‐EDS that Pt is dispersed as a submonolayer. The coexistence of oxidized and metallic Pt species is also indicative of highly exposed and reactive surface Pt sites, which are beneficial for HER catalysis. Although bulk electronegativity values suggest charge transfer from Pt to Au, electron redistribution in Au–Pt nanostructures depends strongly on coverage regime and local coordination. In our system, discontinuous submonolayer Pt decoration creates undercoordinated Pt atoms that favor electron enrichment, as confirmed by DFT Bader charge analysis. This contrasts with alloyed systems, where higher Pt coordination drives Pt → Au transfer.^[^ ^25^ ^]^ Thus, charge redistribution in Au–Pt systems cannot be explained by electronegativity alone, but must be understood in the context of nanoscale morphology.

**Figure 4:**
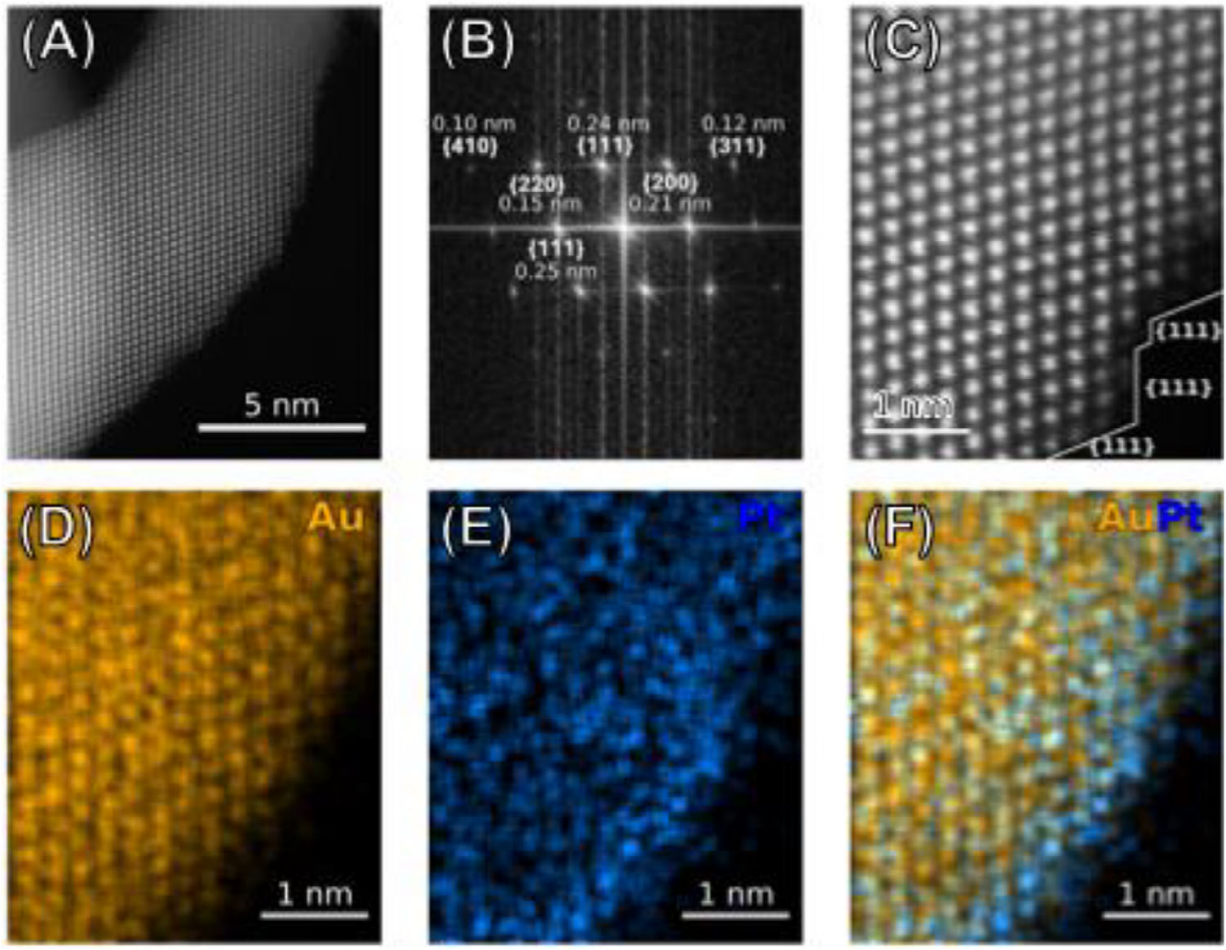
Structural and compositional characterization of Au_92_Pt_8_ nanowires. A) HAADF‐STEM image of an individual nanowire segment, and B) corresponding fast Fourier transform (FFT) pattern showing well‐defined lattice spacings assigned to (111) and (100). C) Atomic‐resolution HAADF‐STEM image showing lattice‐resolved morphology with predominant {111} surface facets. D–F) Corresponding STEM‐EDS elemental maps of the region in (C): (D) Au, (E) Pt, and (F) the Au–Pt overlay. The maps indicate the formation of a continuous 1–2 atomic layer Pt shell over the Au nanowire core.

Regarding the Pt distribution, we emphasize that the conclusion of submonolayer Pt arises from a convergence of complementary evidence: i) STEM‐EDS mapping shows no continuous Pt shell in Au_98.5_Pt_1.5_ nanowires, in contrast to Au_92_Pt_8_ (shown later in **Figure** **4**); ii) atomic‐resolution HAADF‐STEM images reveal faint contrast features corresponding to isolated, mobile surface atoms absent in pure Au nanowires (Figure S10, Supporting Information), strongly suggestive of Pt single atoms or clusters; and iii) XPS confirms the presence of both metallic Pt^0^ and oxidized Pt^2+^, consistent with highly exposed, discontinuous Pt at ultralow loadings. In view of the difficulty in distinguishing Au and Pt atoms unambiguously by STEM alone, we therefore use the conservative term submonolayer distribution.

To further illustrate how Pt coverage influences structural features, we characterized the Au_92_Pt_8_ nanowires, representing the high‐Pt end‐member of our series (Figure 4A–F). In contrast to the discontinuous submonolayer Pt decoration observed for Au_98.5_Pt_1.5_, the Au_92_Pt_8_ sample exhibits a more continuous coverage (1–2 atomic layer Pt shell over the Au nanowire core). HAADF‐STEM imaging (Figure 4A,C; Figure S13, Supporting Information) and FFT analysis (Figure 4B) reveal well‐defined lattice fringes with dominant {111} surface facets similar to those in the Au_98.5_Pt_1.5_ and pure Au nanowires. However, STEM‐EDS mapping reveals that the higher Pt content leads to a more continuous and uniform distribution of Pt over the nanowire surface compared to that seen in Au_98.5_Pt_1.5_ nanowires (Figure 4E,F). This surface coating has a thickness of 0–2 atomic layers in the region imaged in Figure 4 (Figure S14, Supporting Information), similarly showing another region of nanowire with similar layer thicknesses. This comparison highlights the structural evolution with increasing Pt loading and explains the dampening of plasmonic responses at higher Pt contents.

The enhanced HER activity observed for the Au_98.5_Pt_1.5_ nanowires can thus be rationalized by considering both electronic and plasmonic effects. For the submonolayer coverage of Au_98.5_Pt_1.5_, Pt atoms are more discretely distributed on the Au surface, leading to a higher probability of forming more isolated catalytic sites with reduced Pt–Pt coordination to more isolated catalytic sites with lower Pt–Pt coordination numbers.^[^ ^49^ ^]^ While we did not directly measure coordination numbers using EXAFS or related techniques, the inference of lower coordination is supported by atomic‐resolution HAADF‐STEM, XPS, and STEM‐EDS. More isolated Pt sites possess altered electronic states, typically characterized by an upshifted d‐band center, as supported by our DFT calculations, which results in optimized binding interactions with reaction intermediates, such as adsorbed hydrogen (H*).^[^ ^49^, ^50^ ^]^ In contrast, the continuous 1–2 atomic layer Pt shell observed for Au_92_Pt_8_ nanowires increases Pt–Pt coordination, shifting electronic properties closer to bulk‐like Pt and weakening the advantage of electronic synergy between Au and more isolated Pt sites. Furthermore, from a plasmonic perspective, the discrete submonolayer coverage of Pt in the Au_98.5_Pt_1.5_ nanowires preserves the strong LSPR absorption of the Au core, thereby facilitating efficient generation and injection of plasmon‐induced hot electrons into surface Pt sites.^[^ ^51^ ^]^ In contrast, a continuous Pt shell may damp the plasmon resonance more strongly, reducing the plasmon‐driven hot‐carrier generation efficiency, and resulting in lower catalytic activity despite the higher total Pt content. Thus, achieving dispersed Pt species on the surface is proposed to be critical to balancing catalytic activity, plasmonic resonance, and efficient hot‐electron utilization, ultimately maximizing HER performance. However, when the Pt content is further decreased to 1 at.% in the Au_99_Pt_1_ nanowires, a noticeable decline in catalytic activity is observed. This reduction can be attributed to the insufficient availability of Pt active sites for effective electron transfer and hydrogen adsorption. While submonolayer Pt coverage in the Au_98.5_Pt_1.5_ nanowires optimally balances electronic synergy and plasmonic enhancement, reducing the Pt loading further limits the density of accessible catalytic sites, which becomes a critical bottleneck for HER performance. Therefore, while minimizing Pt content is desirable for cost and resource considerations, our results suggest that there exists an optimal range, exemplified by Au_98.5_Pt_1.5_ nanowires, below which further reductions compromise catalytic efficiency due to insufficient availability of catalytically active sites. Our data on Au_99_Pt_1_ nanowires confirmed that this lowest‐Pt loading sample preserved the characteristic nanowire morphology (TEM/SEM, Figure S4, Supporting Information), exhibited a narrow size distribution (Figure S5, Supporting Information), and retained the Au plasmon resonance (UV–vis, Figure S6, Supporting Information). These results demonstrate that the structural framework is consistent across the compositional series, validating Au_99_Pt_1_ as the low‐Pt end‐member. Together with the detailed atomic‐resolution analysis of Au_98.5_Pt_1.5_ and Au_92_Pt_8_, these findings provide sufficient evidence that the evolution of structure with Pt loading is fully captured without requiring redundant high‐resolution characterization for Au_99_Pt_1_.

**Figure 5:**
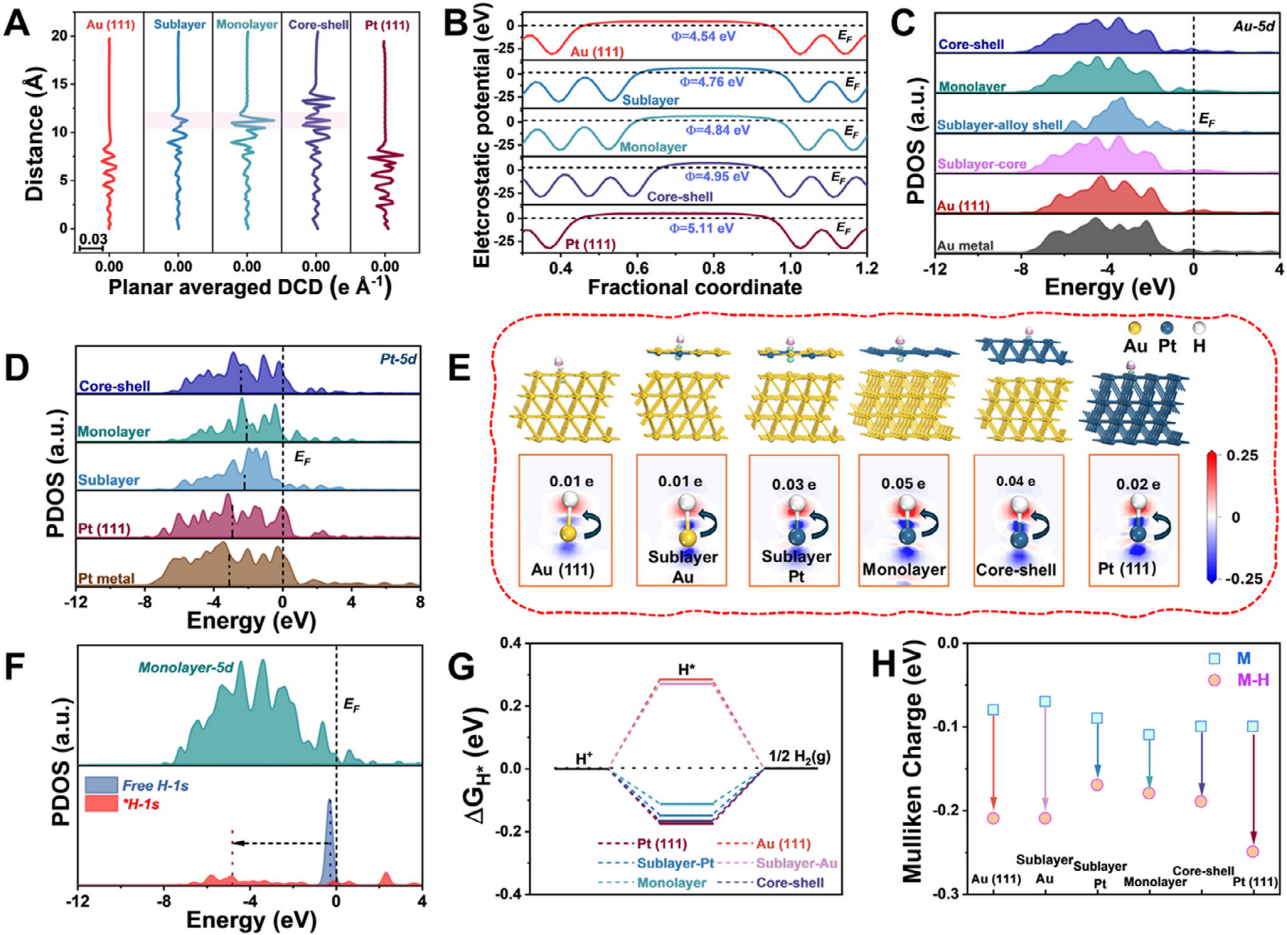
DFT analysis of electronic structure and hydrogen adsorption across Au–Pt surface models. A) Plane‐averaged differential charge density (DCD) profiles along the z‐axis for Au (111), Au‐Pt sublayer, Au‐Pt monolayer, Au‐Pt core–shell, and Pt (111) surface models. B) Electrostatic potential profiles showing progressive changes in work function with increasing Pt coverage. C,D) Projected density of states (PDOS) of (C) Au 5d and (D) Pt 5d orbitals under different coordination environments. E) Charge density difference (CDD) plots and Mulliken charge analysis for H adsorption on various surfaces. Green and pink contours indicate regions of electron accumulation and depletion, respectively (isosurface range: −0.25 to +0.25 e). F) PDOS comparison between free H and H adsorbed at Pt sites in the monolayer model, showing hybridization of the H 1s orbital with surface states. G) Calculated Gibbs free energies for hydrogen adsorption (ΔG_H*_) across all models. H) Mulliken charge analysis before and after H adsorption, highlighting efficient electron transfer at monolayer Pt sites.

To further elucidate the electronic and structural origins of the enhanced HER activity observed for the Au_98.5_Pt_1.5_ nanowires, we performed additional DFT calculations specifically focused on detailed slab models of the (111) surface facet. These calculations differ from the initial DFT analyses (Figure 1), which employed simplified nanowire models including multiple facets and edges, by providing a more precise examination of the electronic interactions and surface phenomena localized exclusively at the dominant catalytic facet. Using surface slab models representing key compositional motifs, pristine Au, Au–Pt sublayer, Au–Pt monolayer, Au–Pt core–shell, and pure Pt surfaces (**Figure** **5**; Figure S15, Supporting Information), allowed us to mimic the experimentally observed configurations and probe the electronic behavior at atomic precision. To assess how surface Pt incorporation alters charge distribution and interfacial behavior, we analyzed the charge density difference (CDD) maps and the corresponding plane‐averaged differential charge density (DCD) profiles along the surface normal (z‐direction) (Figure 5A; Figure S16, Supporting Information). The DCD analysis revealed significant electron redistribution at the metal–metal interface.^[^ ^52^ ^]^ This interfacial polarization, driven by strong electronic coupling between the Au core and Pt atoms at the surface, may establish a built‐in electric field across the interface that facilitates spatial separation and directional transport of plasmon‐generated hot carriers. Electrostatic potential profiles across the models (Figure 5B) further confirmed this behavior, showing a progressive increase in work function (W_F_) with higher Pt content. This trend reflects spontaneous electron migration from the Au core to the more electronegative Pt‐rich surface, aligning the Fermi levels and stabilizing the system.^[^ ^53^ ^]^ The resulting charge equilibration ensures that photogenerated hot electrons accumulate preferentially on the surface Pt sites, precisely where HER takes place, thus enhancing the overall efficiency of plasmon‐driven catalysis.

Next, we examined the projected density of states (PDOS) of Au 5d and Pt 5d orbitals in different configurations (Figure 5C,D, respectively). For the Au 5d states (Figure 5C), Pt decoration induced a downward shift in the density of states, indicative of electron transfer from Au to Pt and a concomitant depopulation of Au surface states. Conversely, the Pt 5d states (Figure 5D) displayed a notable upshift in both the monolayer and sublayer configurations, resulting in elevated d‐band centers relative to bulk Pt and the core–shell model. This shift toward the Fermi level is associated with stronger orbital overlap and enhanced binding of reaction intermediates, a hallmark of improved catalytic activity.^[^ ^53^ ^]^ Among all configurations, the sublayer and monolayer models exhibited the highest d‐band centers, consistent with the theoretically optimal hydrogen adsorption energy. Charge density difference (CDD) mapping and Mulliken population analysis (Figure 5E) revealed significant electron redistribution localized at Pt active sites upon H adsorption, especially in the monolayer model, where pronounced charge accumulation and depletion highlight efficient coupling between the Pt surface and H*.^[^ ^54^ ^]^ Complementary PDOS analysis of the H 1s orbital (Figure 5F) confirmed strong hybridization with Pt states, supporting effective electron transfer during the adsorption process.

The catalytic implications of these interactions were quantified by calculating the Gibbs free energy of hydrogen adsorption (ΔG_H*_, Figure 5G). The Pt sites in the monolayer model exhibited ΔG_H*_ values closest to the thermoneutral condition (≈0 eV),^[^ ^55^ ^]^ suggesting an ideal balance between H* adsorption and desorption. This would, in principle, translate to optimal HER performance. However, experimentally, the best catalytic activity was observed for the Au_98.5_Pt_1.5_ nanowires, which exhibit submonolayer Pt coverage rather than a complete monolayer. This apparent discrepancy can be reconciled by considering the nature of plasmonic enhancement and local heterogeneity in real catalyst surfaces. While DFT calculations assume idealized, ordered surfaces, the experimental submonolayer coverage produces a heterogeneous distribution of Pt atoms—ranging from more isolated atoms to layered regions on the Au surface. Such configurations can maximize the electronic synergy between Au and Pt, creating low‐coordination Pt sites that are known to be catalytically superior.

Interestingly, Mulliken charge analysis (Figure 5H) revealed that Pt sites in the monolayer model experienced the greatest electron loss upon H adsorption, despite stronger H binding predicted by their elevated d‐band centers. This suggests a synergistic interaction between adjacent Au and Pt atoms in mixed environments, facilitating directional charge transfer from the Au scaffold to Pt and then to the H* intermediate.^[^ ^56^ ^]^ The experimentally realized submonolayer architecture captures the best of both regimes: sufficient Pt coverage to provide active sites, and enough surface heterogeneity and electronic coupling to enhance both intrinsic activity and plasmonic responsiveness. Taken together, these insights offer a coherent atomic‐scale explanation for the superior catalytic performance of Au_98.5_Pt_1.5_ NWs. They highlight how submonolayer Pt coverage on anisotropic Au supports can simultaneously optimize hydrogen adsorption thermodynamics and electronic structure, critical factors for maximizing HER activity under light‐driven and precious‐metal‐limited conditions.

In summary, our results establish a clear structure–activity–plasmonic relationship. The anisotropic Au nanowire architecture not only provides high surface accessibility and efficient charge transport but also preserves Au LSPR even at ultralow Pt coverages. This retention of plasmonic activity is critical, as it enables hot‐carrier generation and interfacial charge redistribution at the Au–Pt interface, which in turn optimizes hydrogen adsorption on Pt. The correlation between preserved LSPR features (UV–vis, Figure S6, Supporting Information), favorable electronic coupling (DFT, Figures S1,S2,S18 and S19, Supporting Information), and enhanced HER activity under illumination demonstrates that plasmonic effects play a decisive role in promoting HER efficiency at minimal Pt loading.

## 3 Conclusion

In this work, we established anisotropic AuPt nanowires with submonolayer Pt coverages as an efficient platform for plasmon‐enhanced hydrogen evolution. Guided by DFT calculations, we synthesized nanowires with controlled Pt distributions ranging from 1–2 atomic layers to discontinuous submonolayer decoration. Advanced structural analyses confirmed the preservation of nanowire morphology, predominant {111} facets, and surface Pt decoration, while optical and spectroscopic measurements demonstrated that Au LSPR are retained even at ultralow Pt loadings. Electrochemical studies revealed that these structural features translate into high HER activity, with Au_98.5_Pt_1.5_ nanowires achieving overpotentials as low as 35 mV at −10 mA cm^−2^ and a mass activity of 9.3 A mg^−1^Pt at −0.05 V vs RHE under visible‐light illumination, approximately sevenfold higher than commercial Pt/C under the same conditions. Importantly, long‐term testing confirmed the durability of the catalysts, with nanowire morphology and integrity preserved after 24 h of continuous operation. Together, these results highlight a clear structure–activity–plasmonic relationship: anisotropic nanowire geometry provides open architectures for electrolyte access, submonolayer Pt decoration maximizes Pt utilization efficiency, and plasmonic excitation drives hot‐carrier transfer and interfacial charge redistribution that further enhance catalytic activity. Beyond the specific system studied here, this work establishes a broadly applicable design principle for coupling plasmonic antenna scaffolds with atom‐efficient catalytic reactors, offering a promising strategy to reduce precious‐metal usage while advancing sustainable hydrogen production.

## 4 Experimental Section

### Materials and Methods

The following reagents were used without further purification: gold(III) chloride trihydrate (HAuCl_4_·3H_2_O, 99.9%, Sigma–Aldrich), dipotassium hexachloroplatinate(IV) (K_2_PtCl_6_, 99.9%, Sigma–Aldrich), 1‐hydroxynaphthalene (C_10_H_7_OH, 99%, Sigma–Aldrich), sodium citrate tribasic dihydrate (≥99%, Sigma–Aldrich), L‐ascorbic acid (≥99%, Sigma–Aldrich), nitric acid (HNO_3_, 65%, Sigma–Aldrich), sulfuric acid (H_2_SO_4_, 98%, Sigma–Aldrich), isopropanol (≥99.9%, Sigma–Aldrich), acetone (99%, Honeywell), Nafion perfluorinated resin solution (10 wt.% in H_2_O, Sigma–Aldrich), ethanol (99.8%, Fisher Chemical), and Vulcan XC‐72R carbon black (Fuel Cell Store). Ultrapure water (18.2 MΩ·cm) was obtained using a Milli‐Q system and used for all synthesis and washing steps.

Field‐emission scanning electron microscopy (FE‐SEM) was carried out using a Hitachi S‐4800 operated at 10 kV and a working distance of 8 mm. Samples were prepared by drop‐casting ethanol‐dispersed nanowires onto silicon wafers. Transmission electron microscopy (TEM) was performed on a JEOL JEM‐1400 microscope, with samples prepared by drop‐casting ethanol suspensions onto carbon‐coated copper grids. Particle size distributions were determined by measuring the diameters of 100 particles.

Atomic‐resolution HAADF‐STEM and STEM‐EDX elemental mapping were carried out using a probe‐corrected Thermo Fisher Spectra 200 S/TEM operated at 200 kV, equipped with a ChemiSTEM EDX system. Imaging conditions included a probe current of ≈150 pA, a semi‐convergence angle of 29.5 mrad, and an inner collection angle of 56 mrad. Tomographic analysis was conducted using a Thermo Fisher Spectra 200 S/TEM with a probe current of ≈80 pA, a convergence angle of 11 mrad, and a collection angle of 56 mrad. Tilt‐series were acquired from −54° to 68° (for the Au_98.5_Pt_1.5_) and −60° to 62° (for the Au) with 2° increments. Tomographic reconstruction was completed in Inspect3D using an Expectation Maximization algorithm with 20 iterations.

UV–vis spectra were recorded using a Shimadzu UV–2600 spectrometer over the range 300–1100 nm (1 nm step) from aqueous nanoparticle suspensions. XRD patterns were collected on a Bruker D8 Advance diffractometer using Cu Kα radiation (λ = 1.5406 Å) in Bragg–Brentano geometry. Data were acquired from 20° to 70° 2θ with a step size of 0.02° and 1 s per step. Diffraction patterns were indexed using JCPDS reference files.

Elemental analysis was performed by microwave plasma atomic emission spectroscopy (MP‐AES, Agilent 4100 MP‐AES) with three independent measurements per sample. X‐ray photoelectron spectroscopy (XPS) was carried out using a PREVAC system equipped with a monochromatized Al Kα source (1486.7 eV) under ultrahigh vacuum. The pressure in the chamber during the measurements was ≈3 × 10^−10^ mbar. Survey and high‐resolution spectra were acquired at 200 and 100 eV pass energy, respectively. All spectra were calibrated to the C 1s peak and analyzed using CasaXPS with a Shirley background correction.

### Synthesis of Au and AuPt Nanowires

Au and Au@AuPt nanowires were synthesized following a modified protocol based on Chen et al.^[^ ^20^ ^]^ In a 25 mL round‐bottom flask, 3 mL of ultrapure water, 3 mL of ethanol, 1 mL of HAuCl_4_·3H_2_O (59 mmol), and varying volumes of K_2_PtCl_6_ (2.4 mmol) were mixed to achieve target Pt coverages (core–shell, monolayer, and submonolayer) as summarized in Table S1 (Supporting Information). Then, 1 mL of 1‐hydroxynaphthalene (0.5 m in ethanol) was added as both a reducing and shape‐directing agent. The reaction mixture was heated at 75 °C for 12 h under reflux using a silicone oil bath. After cooling to room temperature, the product was washed seven times with ethanol via centrifugation and resuspended in ethanol for storage. Control Au nanowires were synthesized identically but without the addition of the Pt precursor.

### Electrocatalytic Evaluation: HER

Electrochemical measurements were conducted in a conventional three‐electrode glass cell using an Autolab PGSTAT 128N potentiostat. A glassy carbon electrode (GCE, 6 mm diameter, 0.2827 cm^2^ geometric area) was used as the working electrode, a graphite rod as the counter electrode, and a reversible hydrogen electrode (RHE) as the reference. For the catalyst ink preparation, a 1 mL dispersion of AuPt nanowires (2.32 mg mL^−1^) was centrifuged and the supernatant removed. The solid was redispersed in 1 mL of a solution containing ethanol:water:Nafion 5% in a 7:2.9:0.1 v/v/v ratio. Vulcan XC‐72R carbon was added to yield a final carbon concentration of 1.16 mg mL^−1^. The resulting suspension was ultrasonicated for 60 min to obtain a homogeneous ink. A 24.4 µL aliquot of the freshly prepared ink was drop‐cast onto a glassy carbon electrode (geometric area = 0.2827 cm^2^). The film was dried at 50 °C for 10 min, resulting in final loadings of 200 mg cm^−2^ of Au and 100 mg cm^−2^ of C.

All measurements were carried out in 0.5 m H_2_SO_4_ (Ar‐purged) at room temperature (25 °C). Linear sweep voltammetry (LSV) was performed at 5 mV s^−1^. For light experiments, we used a Kessil PR160L LED (λ = 525 nm, 68 mW cm^−2^, 5 cm distance). Long‐term stability measurements (chronoamperometry), however, were carried out in the dark at a fixed current density of −10 mA cm^−2^, in order to provide a conservative and light‐independent benchmark of electrochemical durability. This approach ensures that catalyst degradation, if present, can be attributed to intrinsic electrochemical processes rather than fluctuations associated with illumination. Mass activities were determined by normalizing the current obtained from LSV to the Pt mass deposited on the electrode. The Pt content of each catalyst was quantified by MP‐AES (Table S1, Supporting Information). Geometrically normalized voltammograms and additional controls are provided in the Supporting Information.

### Theoretical Calculations

DFT calculations were performed using the CASTEP package to investigate the electronic structure and HER activity of Pt‐decorated Au nanowires.^[^ ^57^ ^]^ The exchange‐correlation interactions were described using the Perdew–Burke–Ernzerhof (PBE) functional within the generalized gradient approximation (GGA).^[^ ^58^ ^]^ Ultrasoft pseudopotentials were employed with a plane‐wave cutoff energy of 420 eV. van der Waals interactions were accounted for using Grimme's DFT‐D dispersion correction.^[^ ^59^ ^]^ Geometry optimizations were carried out using the Broyden–Fletcher–Goldfarb–Shanno (BFGS) algorithm until forces converged below 0.05 eV Å^−1^, energy convergence was within 5 × 10^−5^ eV per atom, and atomic displacements were less than 0.005 Å. A vacuum spacing of 20 Å was included along the c‐axis to avoid periodic interactions between surfaces. Simulated models correspond to various Pt coverage scenarios as detailed in the manuscript and Supporting Information.

## Conflict of Interest

The authors declare no conflict of interest.

## Supporting information



Supporting Information

## Data Availability

The data that support the findings of this study are available in the supplementary material of this article.

## References

[1] A. A. Feidenhans'l , Y. N. Regmi , C. Wei , D. Xia , J. Kibsgaard , L. A. King , Chem. Rev. 2024, 124, 5617. 38661498 10.1021/acs.chemrev.3c00712 PMC11082907

[2] F. Liu , C. Shi , X. Guo , Z. He , L. Pan , Z. F. Huang , X. Zhang , J. J. Zou , Adv. Sci. 2022, 9, 2200307. 10.1002/advs.202200307 PMC9218766 35435329

[3] D. Voiry , H. S. Shin , K. P. Loh , M. Chhowalla , Nat. Rev. Chem. 2018, 2, 0105.

[4] N. Mahmood , Y. Yao , J. W. Zhang , L. Pan , X. Zhang , J. J. Zou , Adv. Sci. 2018, 5, 1700464. 10.1002/advs.201700464 PMC5827647 29610722

[5] Z. W. She , J. Kibsgaard , C. F. Dickens , I. Chorkendorff , J. K. Nørskov , T. F. Jaramillo , Science 2017, 355, 6321. 10.1126/science.aad4998 28082532

[6] N. K. Pandit , D. Roy , S. C. Mandal , B. Pathak , J. Phys. Chem. Lett. 2022, 13, 7583. 35950905 10.1021/acs.jpclett.2c01401

[7] Y. Luo , Y. Zhang , J. Zhu , X. Tian , G. Liu , Z. Feng , L. Pan , X. Liu , N. Han , R. Tan , Small Methods 2024, 8, 2400158. 38745530 10.1002/smtd.202400158 PMC11672190

[8] M. A. Qadeer , X. Zhang , M. A. Farid , M. Tanveer , Y. Yan , S. Du , Z. F. Huang , M. Tahir , J. J. Zou , J. Power Sources 2024, 613, 234856.

[9] M. A. Deshmukh , A. Bakandritsos , R. Zbořil , Nanomicro Lett. 2025, 17, 1. 10.1007/s40820-024-01505-2 PMC11422407 39317789

[10] D. Wang , Y. Li , Adv. Mater. 2011, 23, 1044. 21218429 10.1002/adma.201003695

[11] P. H. C. Camargo , E. Cortés , in Plasmonic Catalysis: From Fundamentals to Applications, John Wiley & Sons, Ltd, Hoboken, NJ, USA 2021, pp. 1–331.

[12] Y. Kang , S. M. João , R. Lin , K. Liu , L. Zhu , J. Fu , W. C. Cheong , S. Lee , K. Frank , B. Nickel , M. Liu , J. Lischner , E. Cortés , Nat. Commun. 2024, 15, 198. 38724494 10.1038/s41467-024-47994-y PMC11519563

[13] K. N. da Silva , S. Shetty , S. Sullivan−Allsop , R. Cai , S. Wang , J. Quiroz , M. Chundak , H. L. S. dos Santos , I. M. Abdelsalam , F. E. Oropeza , V. A. de la Peña O'Shea , N. Heikkinen , E. Sitta , T. V. Alves , M. Ritala , W. Huo , T. J. A. Slater , S. J. Haigh , P. H. C. Camargo , ACS Nano 2024, 18, 24391. 39164202 10.1021/acsnano.4c07076 PMC11386439

[14] C. J. Herring , M. M. Montemore , ACS Nano 2025, 19, 9860. 40052953 10.1021/acsnano.4c13602 PMC11924337

[15] J. Quiroz , P. F. M. de Oliveira , S. Shetty , F. E. Oropeza , V. A. Shea , L. C. V. Rodrigues , M. P. Maria , R. M. Torresi , F. Emmerling , P. H. C. Camargo , ACS Sustain. Chem. Eng. 2021, 9, 9750.

[16] W. Ou , Y. Guo , J. Zhong , F. Lyu , J. Shen , H. Li , S. Zhang , Z. Li , Z. He , J. He , Q. Mo , C. Zhi , Y. Y. Li , J. Lu , Energy Environ. Sci. 2025, 18, 1673.

[17] F. Chang , S. Shan , V. Petkov , Z. Skeete , A. Lu , J. Ravid , J. Wu , J. Luo , G. Yu , Y. Ren , C. J. Zhong , J. Am. Chem. Soc. 2016, 138, 12166. 27617338 10.1021/jacs.6b05187

[18] Q. L. Zhu , Q. Xu , Chem 2016, 1, 220.

[19] L. S. Bezerra , P. Brasseur , S. Sullivan‐Allsop , R. Cai , K. N. da Silva , S. Wang , H. Singh , A. K. Yadav , H. L. S. Santos , M. Chundak , I. Abdelsalam , V. J. Heczko , E. Sitta , M. Ritala , W. Huo , T. J. A. Slater , S. J. Haigh , P. H. C. Camargo , Angew. Chem., Int. Ed. 2024, 63, 202405459. 10.1002/anie.202405459 38711309

[20] Q. Xue , X. Y. Bai , Y. Zhao , Y. N. Li , T. J. Wang , H. Y. Sun , F. M. Li , P. Chen , P. Jin , S. B. Yin , Y. Chen , J. Energy Chem. 2021, 65, 94.

[21] J. Solla‐Gullón , F. J. Vidal‐Iglesias , J. M. Feliu , Ann. Rep. Prog. Chem. C 2011, 107, 263.

[22] K. J. Wu , E. C. M. Tse , C. Shang , Z. Guo , Prog. Mater. Sci. 2022, 123, 100821.

[23] T. Wasiak , D. Janas , J. Alloys Compd. 2022, 892, 162158.

[24] H. Xu , H. Shang , C. Wang , Y. Du , Adv. Funct. Mater. 2020, 30, 2000793.

[25] H. Shi , T. Wang , Z. Lin , S. Liu , X. Liu , R. Zhou , Z. Cai , Y. Huang , Q. Li , Angew. Chem., Int. Ed. 2025, 64, 202424476. 10.1002/anie.202424476 39829348

[26] Y. Lee , J. Kim , D. S. Yun , Y. S. Nam , Y. Shao‐Horn , A. M. Belcher , Energy Environ. Sci. 2012, 5, 8328. 24910712 10.1039/C2EE21156D PMC4045645

[27] K. Wei , H. Lin , X. Zhao , Z. Zhao , N. Marinkovic , M. Morales , Z. Huang , L. Perlmutter , H. Guan , C. Harris , M. Chi , G. Lu , K. Sasaki , S. Sun , J. Am. Chem. Soc. 2023, 145, 19076. 37606196 10.1021/jacs.3c07027

[28] B. Y. Xia , H. B. Wu , Y. Yan , X. W. (David) Lou , X. Wang , J. Am. Chem. Soc. 2013, 135, 9480. 23742152 10.1021/ja402955t

[29] W. T. Fan , Y. Qin , X. B. Hu , J. Yan , W. T. Wu , Y. L. Liu , W. H. Huang , Anal. Chem. 2020, 92, 15639. 33179904 10.1021/acs.analchem.0c04015

[30] W.‐Y. Hu , Q.‐Y. Li , G.‐Y. Zhai , Y.‐X. Lin , X. Lin , D. Xu , L.‐H. Sun , S.‐N. Zhang , J.‐S. Chen , X.‐H. Li , D. Li , X.‐X. He , Small 2022, 18, 2200885. 10.1002/smll.202200885 35396794

[31] S. Chavez , U. Aslam , S. Linic , ACS Energy Lett. 2018, 3, 1590.

[32] M. Chen , Z. Ye , L. Wei , J. Yuan , L. Xiao , J. Am. Chem. Soc. 2022, 144, 12842. 35802866 10.1021/jacs.2c04202

[33] D. Wei , Y. Wang , C. L. Dong , T. T. T. Nga , H. Zhou , C. Hu , Y. Shi , J. Wang , L. Guo , S. Shen , Angew. Chem., Int. Ed. 2025, 64, 202512179. 10.1002/anie.202512179 40823978

[34] E. R. Newmeyer , Y. Wang , Z. A. Long , J. D. North , Y. Shi , D. F. Swearer , J. Am. Chem. Soc. 2025, 147, 11789. 40165739 10.1021/jacs.4c14842

[35] K. L. Zhou , C. B. Han , Z. Wang , X. Ke , C. Wang , Y. Jin , Q. Zhang , J. Liu , H. Wang , H. Yan , Adv. Sci. 2021, 8, 2100347. 10.1002/advs.202100347 PMC8224416 34194948

[36] Q. Wen , J. Duan , W. Wang , D. Huang , Y. Liu , Y. Shi , J. K. Fang , A. Nie , H. Li , T. Zhai , Angew. Chem., Int. Ed. 2022, 61, 202206077. 10.1002/anie.202206077 35730919

[37] Z. Luo , Y. Guo , C. He , Y. Guan , L. Zhang , Y. Li , Q. Zhang , C. He , X. Sun , X. Ren , Angew. Chem., Int. Ed. 2024, 63, 202405017. 10.1002/anie.202405017 38749917

[38] L. Zeng , Y. Chen , M. Sun , Q. Huang , K. Sun , J. Ma , J. Li , H. Tan , M. Li , Y. Pan , Y. Liu , M. Luo , B. Huang , S. Guo , J. Am. Chem. Soc. 2023, 145, 17577. 37253225 10.1021/jacs.3c02570

[39] R. Mendoza‐Cruz , L. Bazán‐Díaz , J. J. Velázquez‐Salazar , G. Plascencia‐Villa , D. Bahena‐Uribe , J. Reyes‐Gasga , D. Romeu , G. Guisbiers , R. Herrera‐Becerra , M. José‐Yacamán , Nano Lett. 2016, 16, 1568. 26849249 10.1021/acs.nanolett.5b04184

[40] E. C. Cho , P. H. C. Camargo , Y. Xia , Adv. Mater. 2010, 22, 744. 20217782 10.1002/adma.200903097

[41] E. Cortés , L. V. Besteiro , A. Alabastri , A. Baldi , G. Tagliabue , A. Demetriadou , P. Narang , ACS Nano 2020, 14, 16202. 33314905 10.1021/acsnano.0c08773

[42] P. K. Jain , J. Phys. Chem. C 2019, 123, 24347.

[43] L. Zhou , D. F. Swearer , C. Zhang , H. Robatjazi , H. Zhao , L. Henderson , L. Dong , P. Christopher , E. A. Carter , P. Nordlander , N. J. Halas , Science 2018, 362, 69. 30287657 10.1126/science.aat6967

[44] H. Jung , S. Choung , J. W. Han , Nanoscale Adv. 2021, 3, 6797. 36132358 10.1039/d1na00606a PMC9417748

[45] J. N. Hansen , H. Prats , K. K. Toudahl , N. Mørch Secher , K. Chan , J. Kibsgaard , ACS Energy Lett. 2021, 6, 1175. 34056107 10.1021/acsenergylett.1c00246 PMC8155388

[46] C. C. L. McCrory , S. Jung , I. M. Ferrer , S. M. Chatman , J. C. Peters , T. F. Jaramillo , J. Am. Chem. Soc. 2015, 137, 4347. 25668483 10.1021/ja510442p

[47] R. K. Leary , A. Kumar , P. J. Straney , S. M. Collins , S. Yazdi , R. E. Dunin‐Borkowski , P. A. Midgley , J. E. Millstone , E. Ringe , J. Phys. Chem. C 2016, 120, 20843. 10.1021/acs.jpcc.6b02103 PMC5036133 27688821

[48] C. H. Choi , M. Kim , H. C. Kwon , S. J. Cho , S. Yun , H. T. Kim , K. J. J. Mayrhofer , H. Kim , M. Choi , Nat. Commun. 2016, 7, 10922. 26952517 10.1038/ncomms10922 PMC4786782

[49] A. Trembułowicz , A. Sabik , L. Jurczyszyn , Sci. Rep. 2022, 12, 3859. 35264635 10.1038/s41598-022-07617-2 PMC8907180

[50] Y. Shi , Z. R. Ma , Y. Y. Xiao , Y. C. Yin , W. M. Huang , Z. C. Huang , Y. Z. Zheng , F. Y. Mu , R. Huang , G. Y. Shi , Y. Y. Sun , X. H. Xia , W. Chen , Nat. Commun. 2021, 12, 3021. 34021141 10.1038/s41467-021-23306-6 PMC8140142

[51] L. Zhou , Q. Huang , Y. Xia , Chem. Rev. 2024, 124, 8597. 38829921 10.1021/acs.chemrev.4c00165 PMC11273350

[52] Z. Chen , M. Yang , Y. Li , W. Gong , J. Wang , T. Liu , C. Zhang , S. Hou , G. Yang , H. Li , Y. Jin , C. Zhang , Z. Tian , F. Meng , Y. Cui , Nat. Commun. 2025, 16, 418. 39762329 10.1038/s41467-025-55854-6 PMC11704302

[53] Y. Wang , J. Hu , T. Ge , F. Chen , Y. Lu , R. Chen , H. Zhang , B. Ye , S. Wang , Y. Zhang , T. Ma , H. Huang , Adv. Mater. 2023, 35, 2302538. 10.1002/adma.202302538 37120752

[54] X. Zhang , Y. Yang , Y. Liu , Z. Jia , Q. Wang , L. Sun , L. C. Zhang , J. J. Kruzic , J. Lu , B. Shen , Adv. Mater. 2023, 35, 2303439. 10.1002/adma.202303439 37279880

[55] T. Zhu , J. Huang , B. Huang , N. Zhang , S. Liu , Q. Yao , S. C. Haw , Y. C. Chang , C. W. Pao , J. M. Chen , Q. Shao , Z. Hu , Y. Ma , X. Huang , Adv. Energy Mater. 2020, 10, 2002860.

[56] C. Hu , K. Yue , J. Han , X. Liu , L. Liu , Q. Liu , Q. Kong , C. W. Pao , Z. Hu , K. Suenaga , D. Su , Q. Zhang , X. Wang , Y. Tan , X. M. Huang , Sci. Adv. 2023, 9, eadf9144. 37713495 10.1126/sciadv.adf9144 PMC10881077

[57] S. J. Clark , M. D. Segall , C. J. Pickard , P. J. Hasnip , M. I. J. Probert , K. Refson , M. C. Payne , Zeitschrift fur Kristallographie 2005, 220, 567.

[58] J. P. Perdew , K. Burke , M. Ernzerhof , Phys. Rev. Lett. 1996, 77, 3865. 10062328 10.1103/PhysRevLett.77.3865

[59] S. Grimme , J. Antony , S. Ehrlich , H. Krieg , J. Chem. Phys. 2010, 132, 154104. 20423165 10.1063/1.3382344

