# Supplementary material for: Plasmonically Enhanced Hydrogen Evolution on Anisotropic AuPt Nanowires with Submonolayer Pt Surface Coverage
Source: Small. 2025 Oct 30;21(50):e10990. doi: 10.1002/smll.202510990 (PMC12710118; doi:10.1002/smll.202510990)
Supplement: Supplementary file 1 — Supporting Information [file SMLL-21-e10990-s001.pdf]

NANO · MICRO
small

Supporting Information

for *Small*, DOI 10.1002/smll.202510990

Plasmonically Enhanced Hydrogen Evolution on Anisotropic AuPt Nanowires with Submonolayer Pt Surface Coverage

*IbrahiM Abdelsalam, Shiqi Wang, Hugo L. S. Santos, Ella Kitching, Pranava Pakala, Mykhailo Chundak, Mikko Ritala, Sarah J. Haigh, Thomas J. A. Slater and Pedro H. C. Camargo**

Supporting Information for

Plasmonically Enhanced Hydrogen Evolution on Anisotropic AuPt Nanowires with Submonolayer Pt Surface Coverage

Ibrahim Abdelsalam,[a,†] Shiqi Wang,[a,†] Hugo L. S. Santos,[a] Ella Kitching,[b] Pranava Pakala,[a] Mykhailo Chundak,[a] Mikko Ritala,[a] Sarah J. Haigh,[c] Thomas J. A. Slater,[b] Pedro H. C. Camargo[a,*]

[a]*Department of Chemistry, University of Helsinki, A.I. Virtasen aukio 1, PO Box 55, FIN-0014 Helsinki, Finland*

[b]*Cardiff Catalysis Institute, School of Chemistry, Cardiff University, Cardiff CF10 3AT, United Kingdom*

[c]*Department of Materials, University of Manchester, Manchester M13 9PL, United Kingdom*

**Corresponding author: pedro.camargo@helsinki.fi*

[†]*These two authors contributed equally to this work.*

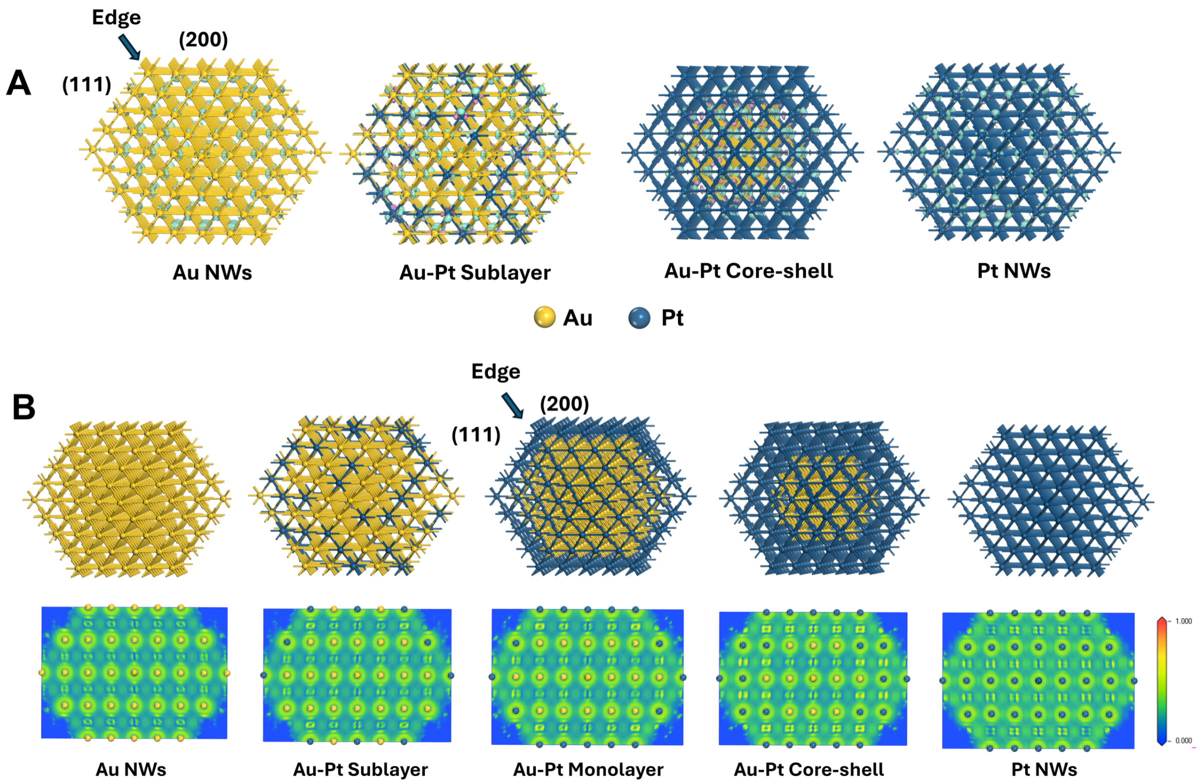

Figure S1. (A) Atomic models and corresponding charge density difference (CDD) maps for Au nanowires (NWs), and AuPt NWs with sublayer, monolayer, and core–shell configurations, as well as pure Pt NWs. Exposed facets include (111) and (200) surfaces. Green and pink isosurfaces indicate regions of electron accumulation and depletion, respectively (isosurface value = 0.05 e Å^{-3}). (B) Structural models and electron localization function (ELF) analysis of Au–Pt nanowires. Top: Atomic models of Au nanowires (Au NWs), Au–Pt sublayer, Au–Pt monolayer, Au–Pt core–shell, and Pt nanowires (Pt NWs) highlighting exposed (111), (200), and edge sites. Bottom: Corresponding two-dimensional electron localization function (ELF) maps projected along the (111) plane. ELF values range from 0 (fully delocalized electrons) to 1 (fully localized electrons). The ELF provides insight into bonding character, with values near 0.5 indicative of electron-gas-like behavior and values near 1 reflecting strong electron localization. The maps reveal variations in electronic delocalization across different Pt distributions, influencing catalytic activity.

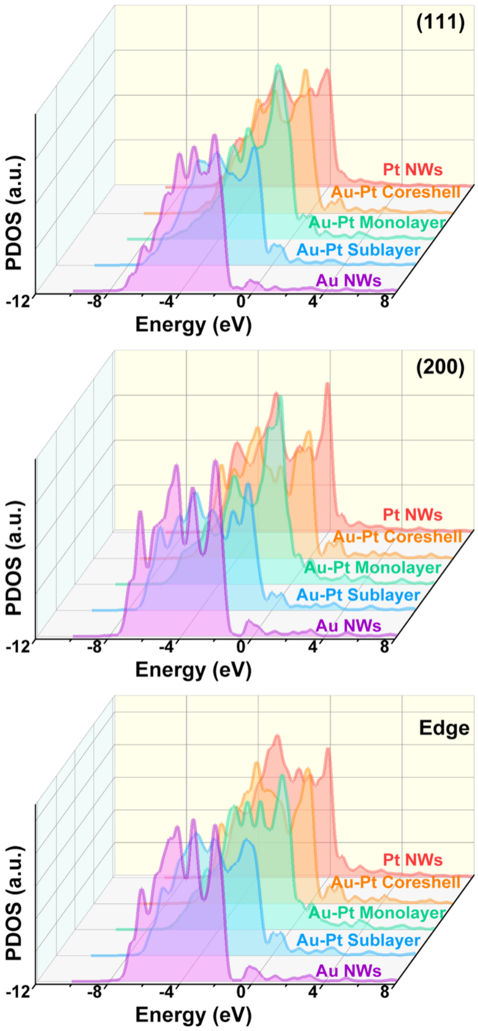

Figure S2. d-band projected density of states (PDOS) for surface and edge sites in Au-Pt nanowire models. Partial density of states (PDOS) for the d orbitals of surface atoms located on (111), (200), and edge sites for five representative models: Au nanowires (Au NWs), Au–Pt sublayer, Au–Pt monolayer, Au–Pt core–shell, and Pt nanowires (Pt NWs). These results highlight the sensitivity of the electronic structure to both surface coordination and Pt distribution. A systematic upshift in the d-band center is observed with increasing Pt content and lower coordination environments, particularly in monolayer and core–shell configurations, suggesting enhanced hydrogen binding affinity relevant to HER activity.

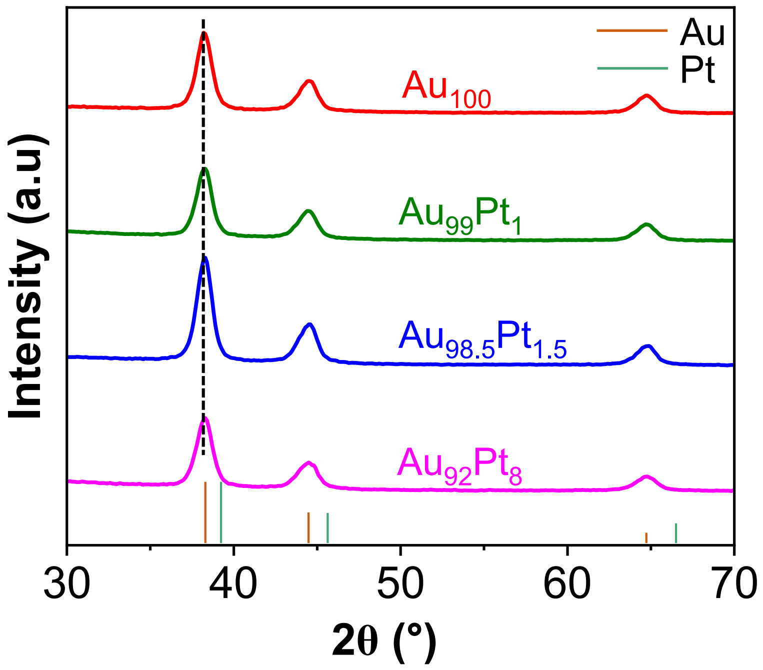

Figure S3. **X-ray diffraction (XRD) patterns of AuPt nanowires with varying Pt surface coverage.** XRD patterns for Au and AuPt nanowires with increasing Pt content: Au_{100}, $Au_{99}Pt_1$, $Au_{98.5}Pt_{1.5}$, and $Au_{92}Pt_8$. All samples exhibit diffraction peaks characteristic of a face-centered cubic (fcc) Au lattice. Reference patterns for pure Au (JCPDS 04-0784) and Pt (JCPDS 04-0802) are included for comparison.

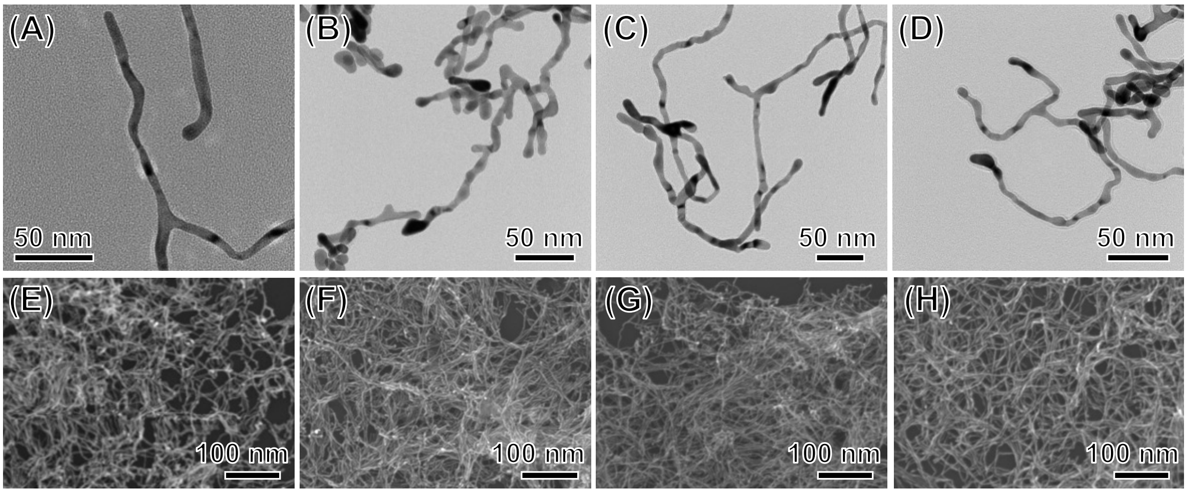

Figure S4. Morphological characterization of AuPt nanowires with varying Pt content. (A-D) TEM and (E-H) SEM images for Au_{100} (A and E), $Au_{92}Pt_{8}$, (B and F), $Au_{98.5}Pt_{1.5}$ (C and G), and $Au_{99}Pt_{1}$ (D and H). All samples exhibit consistent undulating nanowire morphology with no significant changes in structural integrity across different Pt loadings. The TEM images (A–D) confirm the preservation of nanowire shape and dimensions upon Pt incorporation, while the SEM images (E–H) reveal entangled nanowire networks with open architectures, facilitating electrolyte accessibility and electron transport. These consistent morphological features ensure that catalytic performance differences arise primarily from variations in Pt surface coverage and the resulting electronic properties rather than geometry.

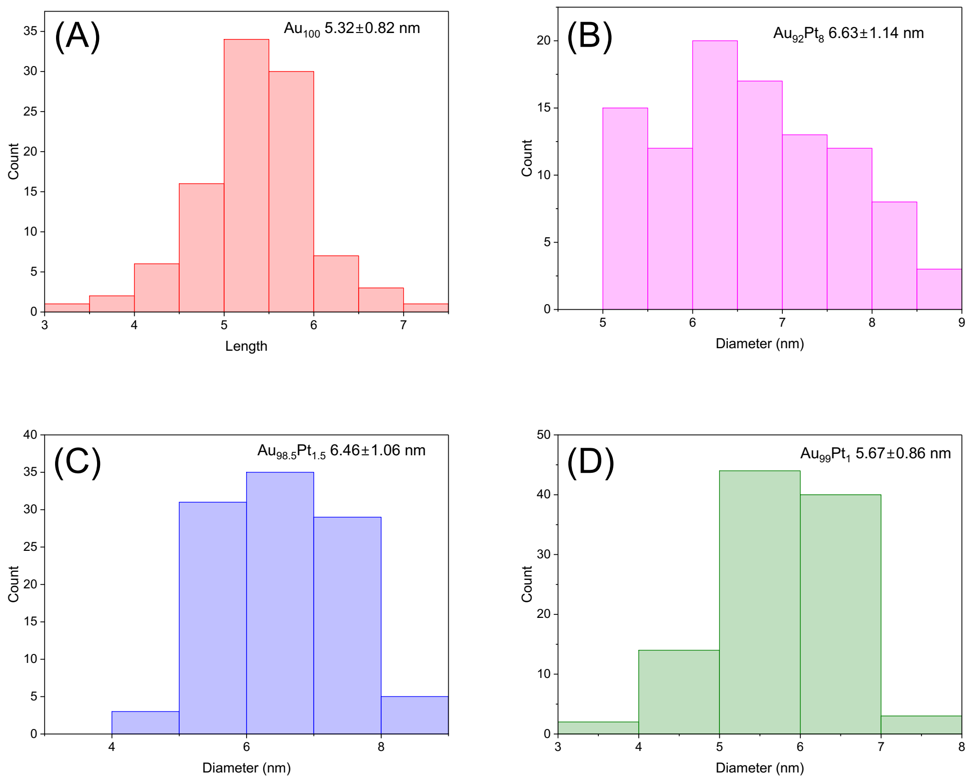

Figure S5. **Size distribution of AuPt nanowires with varying Pt content.** Histograms showing the diameter distributions of Au nanowires (Au_{100}), $Au_{92}Pt_{8}$ $Au_{98.5}Pt_{1.5}$, and $Au_{99}Pt_{1}$. Diameters were measured from TEM images of 100 nanowires for each sample. The data indicate relatively uniform size distributions across all compositions, confirming that Pt incorporation does not significantly alter the overall nanowire dimensions.

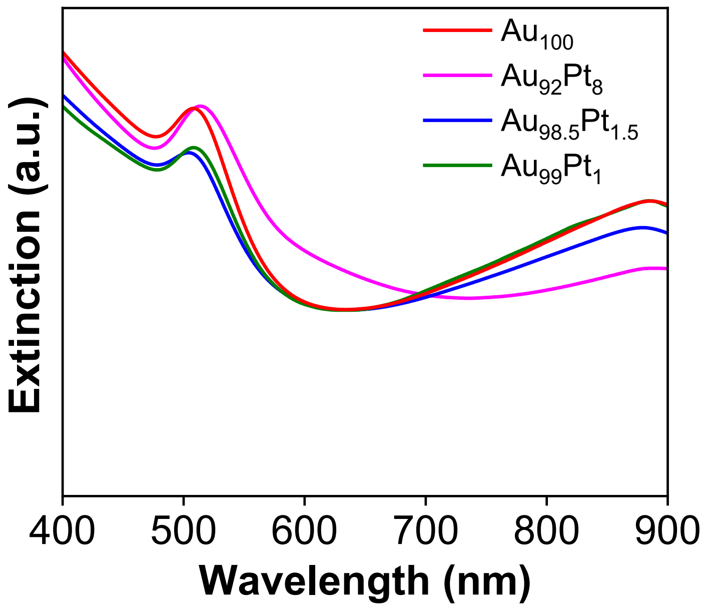

Figure S6. UV–vis extinction spectra of AuPt nanowires with varying Pt content. UV–vis spectra for Au and AuPt nanowires with increasing Pt content: Au_{100}, $Au_{99}Pt_1$, $Au_{98.5}Pt_{1.5}$, and $Au_{92}Pt_8$. All samples exhibit characteristic localized surface plasmon resonance (LSPR) peaks in the visible region near 530 nm, associated with Au nanowire cores. Progressive broadening and slight damping of the plasmon band are observed with increasing Pt incorporation, particularly for $Au_{92}Pt_8$, due to Pt surface decoration.

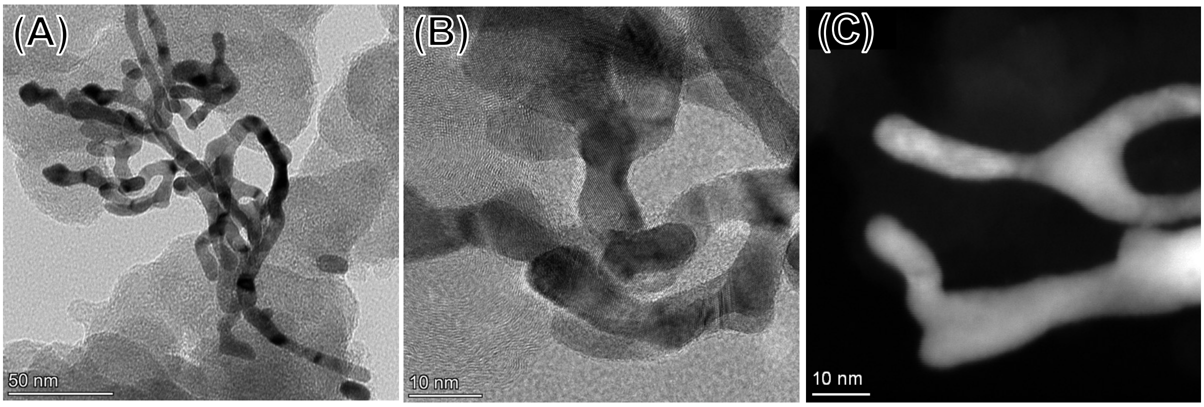

Figure S7. Post-stability electron microscopy analysis of $Au_{98.5}Pt_{1.5}$ nanowires. (A) TEM, (B) HRTEM, and (C) HAADF-STEM images of $Au_{98.5}Pt_{1.5}$ nanowires supported on Vulcan carbon after chronoamperometry testing for 24 hours at −10 mA cm^{-2}. The nanowires retained their morphology and structural integrity, with no detectable aggregation, fragmentation, or morphological deformation, confirming the high stability of the catalyst under prolonged electrochemical operation.

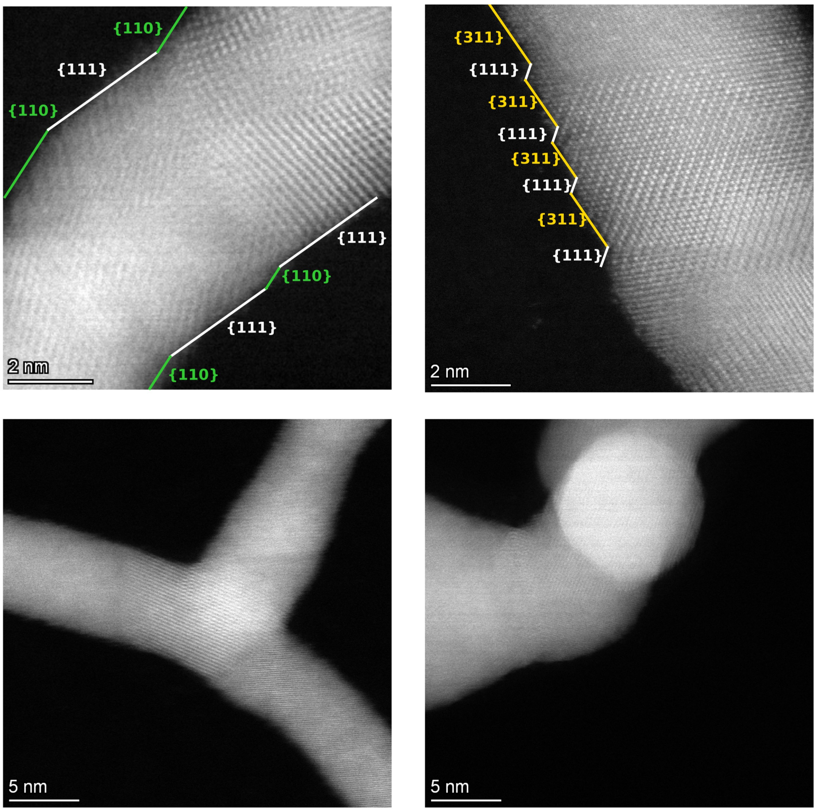

Figure S8. Additional HAADF-STEM images of $Au_{98.5}Pt_{1.5}$ nanowires. Representative HAADF-STEM images of $Au_{98.5}Pt_{1.5}$ nanowires at different magnifications. The images demonstrate the morphology, crystalline lattice fringes, and defective fcc structure of the nanowires, with clear evidence of {111} surface facets. The fcc lattice planes persist to the edges of the nanowires and no additional crystallites are observed on the surfaces, demonstrating that the Pt is coherent with the Au lattice.

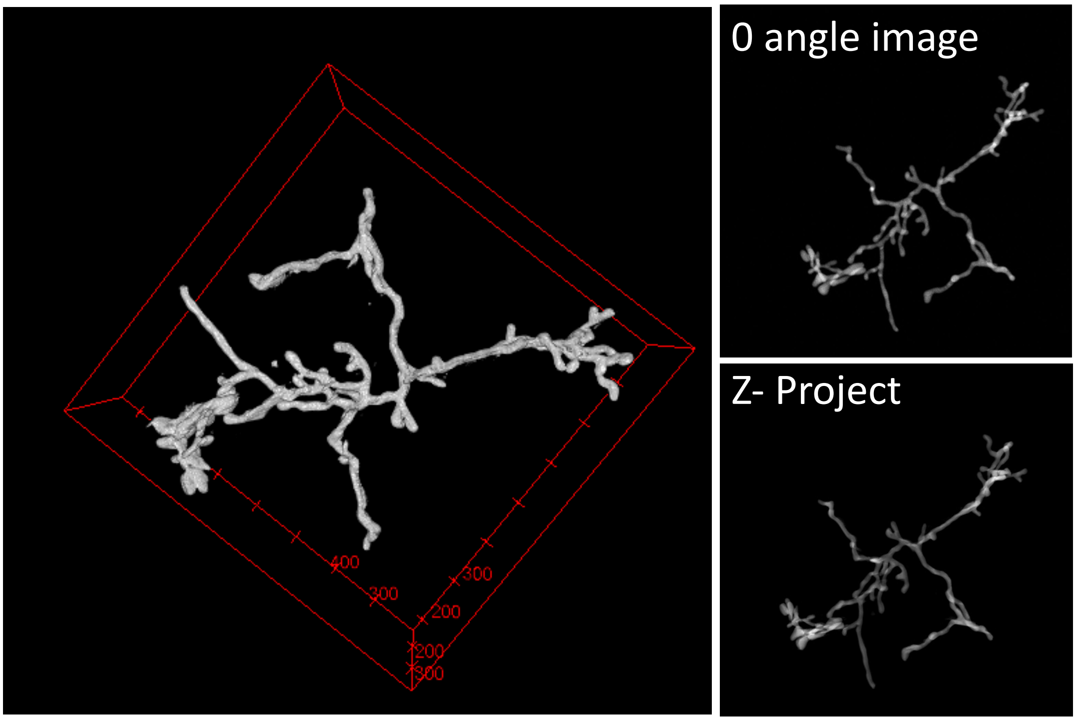

Figure S9. Three-dimensional electron tomography of $Au_{98.5}Pt_{1.5}$ nanowires. 3D reconstruction (left) and corresponding 2D projections (right) of $Au_{98.5}Pt_{1.5}$ nanowires obtained via HAADF-STEM tomography. The 3D rendering reveals a highly interconnected network of nanowires with an open architecture. The 0° tilt angle image (top right) and Z-projection (bottom right) illustrate the preserved morphology from different viewing perspectives, confirming the robustness of the nanowire structure in three dimensions. This open network facilitates electrolyte accessibility and charge transport, contributing to the enhanced catalytic performance and stability of the nanowires during HER operation.

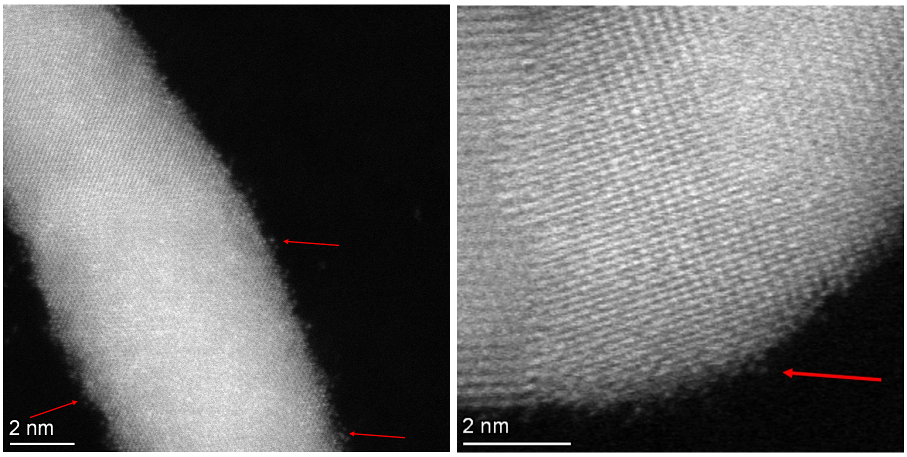

Figure S10. Atomic-resolution HAADF-STEM imaging of $Au_{98.5}Pt_{1.5}$ nanowires showing surface atomic mobility. High-angle annular dark-field scanning transmission electron microscopy (HAADF-STEM) images of $Au_{98.5}Pt_{1.5}$ nanowires at atomic resolution. The core lattice remains well-ordered, while faint contrast features at the nanowire surface (indicated by red arrows) suggest the presence of mobile surface atoms. These features may correspond to isolated Pt single atoms or small atomic clusters not resolved in bulk imaging, consistent with the submonolayer Pt decoration inferred from STEM-EDS and XPS analysis. This surface atomic mobility likely plays a critical role in enhancing catalytic activity by providing dynamic, low-coordination active sites.

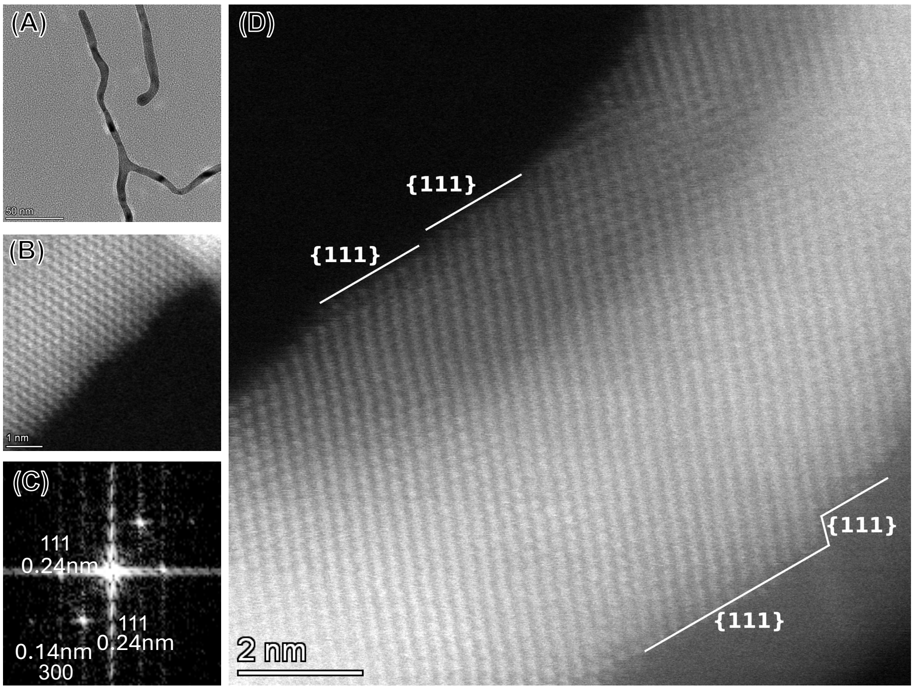

Figure S11 Structural characterization and facet analysis of Au nanowires (control sample). (A) TEM image of pristine Au nanowires (Au_{100}), showing the characteristic morphology. (B) Atomic-resolution HAADF-STEM image revealing well-ordered lattice fringes and stacking defects. (C) Fast Fourier transform (FFT) of the image in (B), with assigned d-spacings of 0.24 nm and 0.14 nm, corresponding to {111} and {200} planes, respectively. (D) Atomic-resolution HAADF-STEM image highlighting facet exposure along the nanowire surface, confirming the predominance of {111} facets with minor presence of {200} facets. These structural features are consistent with those observed in the Pt-decorated Au nanowires and support the role of morphology and facet orientation in catalytic performance.

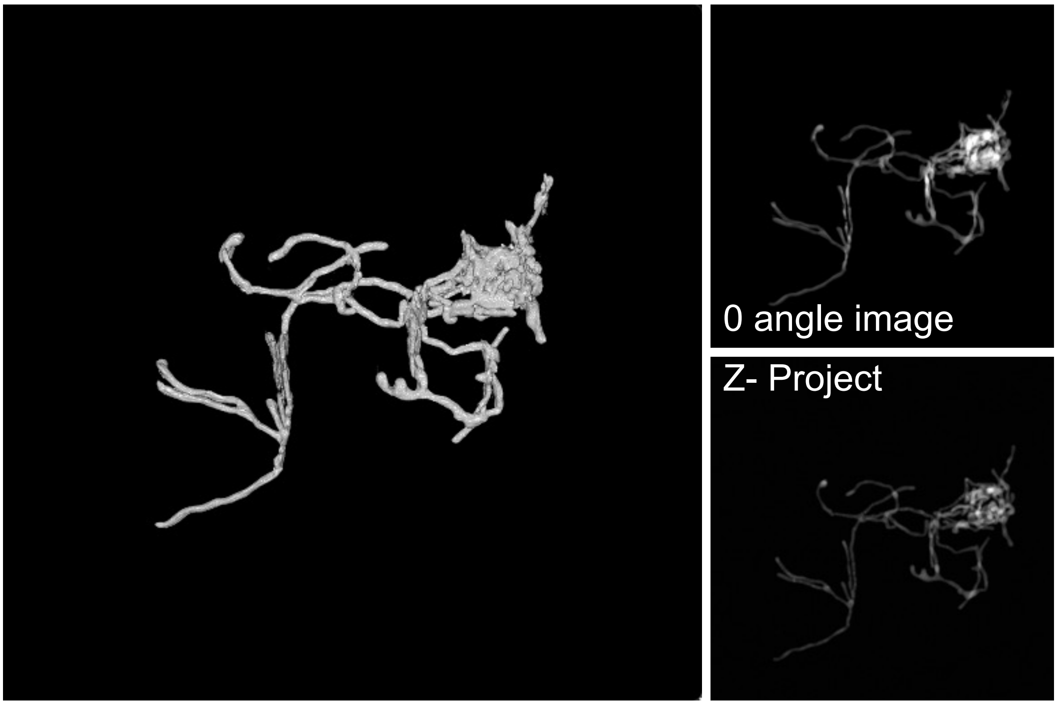

Figure S12. Three-dimensional electron tomography of Au nanowires (control sample). 3D reconstruction (left, see Supporting Movie S2) and corresponding 2D projections (right) of pristine Au nanowires obtained via HAADF-STEM tomography. The reconstruction reveals an interconnected network of nanowires with consistent diameters. The 2D projections illustrate the network morphology from different perspectives, confirming the structural integrity and open framework of the Au nanowires. These morphological features are comparable to those of the Pt-decorated Au nanowires, confirming that Pt incorporation does not significantly alter the overall geometry, allowing direct comparison of catalytic properties based on surface composition rather than morphology.

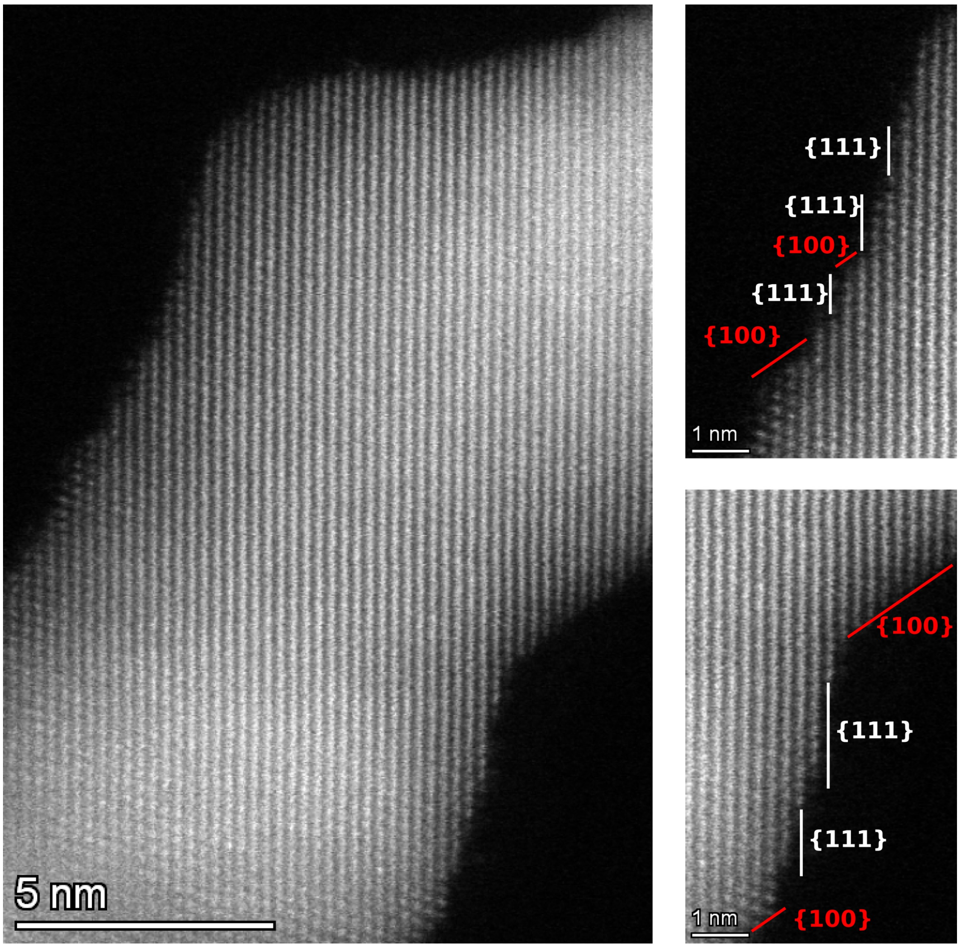

Figure S13. Atomic-resolution HAADF-STEM imaging and facet analysis of $Au_{92}Pt_8$ nanowires. High-angle annular dark-field scanning transmission electron microscopy (HAADF-STEM) images of $Au_{92}Pt_8$ nanowires at atomic resolution, highlighting the crystalline structure and facet orientation. The main panel (left) shows the well-ordered lattice of the nanowire body, while the zoomed-in regions (right) provide detailed facet analysis. Predominantly exposed {111} facets are observed, with minor contributions from {100} facets at specific regions along the nanowire surface.

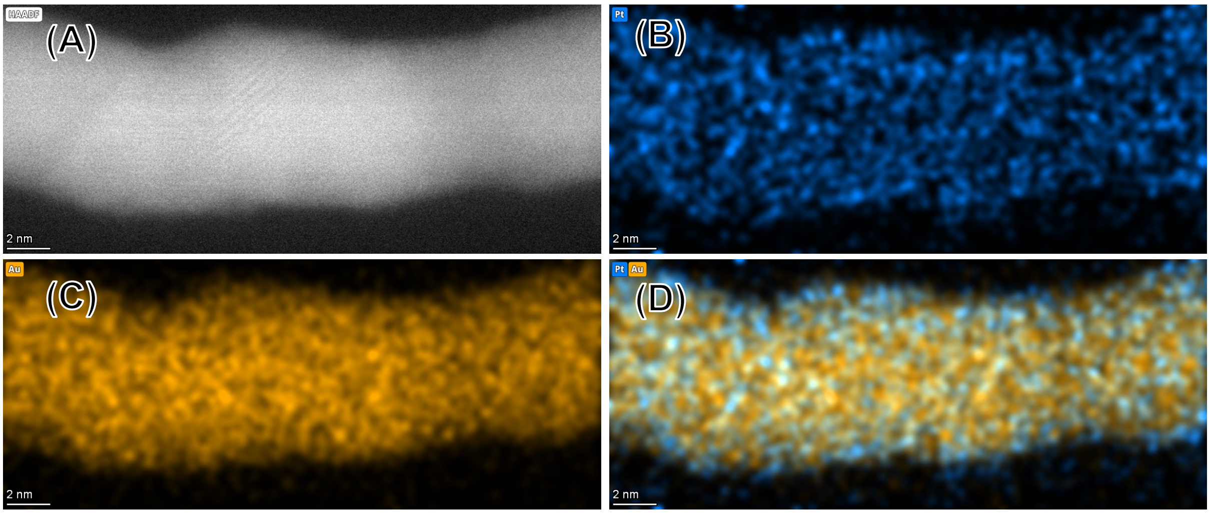

Figure S14. STEM-EDS elemental mapping of $Au_{92}Pt_8$ nanowires illustrating Pt surface distribution. (A) HAADF-STEM image of a representative $Au_{92}Pt_8$ nanowire. (B) Corresponding EDS map for Pt (blue), (C) EDS map for Au (orange), and (D) overlay of Au and Pt distributions. The Pt signal is localized predominantly at the nanowire surface, forming a discontinuous shell approximately 1–2 atomic layers thick, consistent with the intended thin-layer Pt decoration.

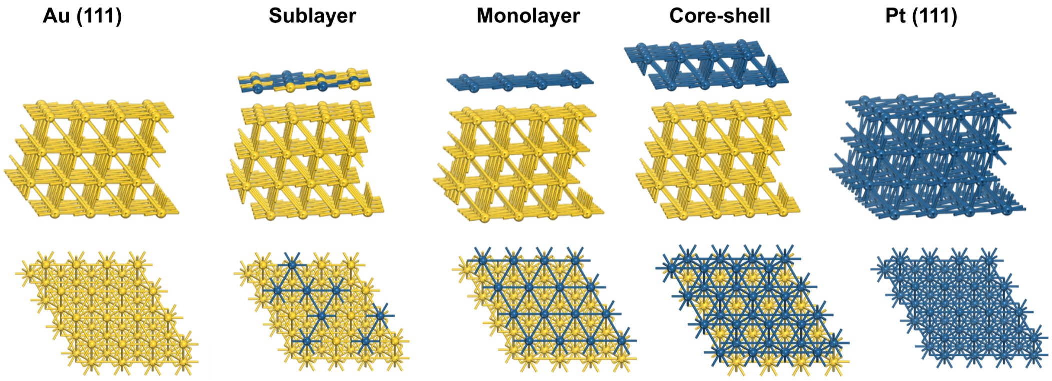

Figure S15. Structural models of Au–Pt surfaces used in DFT calculations. Side and top views of the (111) surface slab models constructed for DFT simulations: pristine Au, Au with sublayer Pt, Au with monolayer Pt, Au with a core–shell Pt overlayer, and bulk Pt. These models were used to evaluate the influence of Pt surface coverage and distribution on the electronic structure and hydrogen adsorption energetics. Au atoms are shown in yellow and Pt atoms in blue.

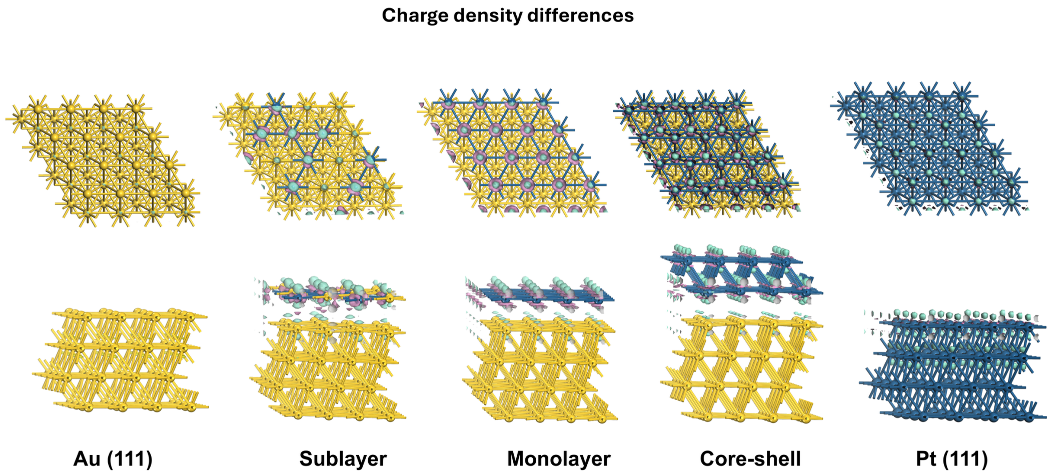

Figure S16. Charge density difference (CDD) analysis of Au–Pt surface models. Top and side views of the charge density difference (CDD) maps for Au (111), Au–Pt sublayer, Au–Pt monolayer, Au–Pt core–shell, and Pt (111) surfaces. Green and purple isosurfaces represent regions of electron accumulation and depletion, respectively, illustrating interfacial charge redistribution upon Pt incorporation. The monolayer and submonolayer configurations exhibit pronounced interfacial polarization between Au and Pt, indicative of strong electronic coupling, whereas the core–shell and pure Pt surfaces display more uniform charge distributions. These results highlight the role of surface Pt arrangement in modulating interfacial electronic structure and enhancing catalytic activity.

Table S1. Measured Au and Pt atomic percentages determined by MP-AES. Reported sample names (e.g., $Au_{98.5}Pt_{1.5}$) are simplified labels used consistently throughout the text and figures for clarity. Exact MP-AES values are provided in the table and should be considered the quantitative reference. The assignment of Pt coverage regimes (submonolayer vs. thin-shell) is based on complementary structural (HAADF-STEM, STEM-EDS, TEM), spectroscopic (XPS), and optical (UV–vis) analyses.

Sample	Au at. %	Pt at. %
Au	100	0
$Au_{92}Pt_8$	92	8.0
$Au_{98.5}Pt_{1.5}$	98.4	1.6
$Au_{99}Pt_1$	99	1.0

Table S2. Comparison of mass activity values obtained in this work with reported Pt-based catalysts, including Pt single-atom and Pt multimetallic systems, measured at –0.10 V in 0.5 M H_2SO_4.

Catalyst	Mass activity (A $mg^{-1}{}_{Pt}$)	Reference
$Au_{98.5}Pt_{1.5}$ (dark)	9.76	This work
$Au_{98.5}Pt_{1.5}$ (525 nm)	15.68	This work
$Au_{92}Pt_8$ (dark)	1.84	This work
$Au_{92}Pt_8$ (525 nm)	2.58	This work
$Au_{99.4}Pt_{0.6}$ (Dark)	6.1	Angew. Chem, 136, e202405459, 2024
$Au_{99.4}Pt_{0.6}$ (525 nm)	7	Angew. Chem, 136, e202405459, 2024
Pt SAs on N–GNs	10.1	Nat Commun 7, 13638, 2016
Pt–Au–SiNW−2	1.8	J. Mater. Chem. A, 5, 21903, 2017
Pt clusters on TiO_2	8.7	Energy Environ. Sci., 10, 2450, 2017
Pt_2Ir_1/CoP	110.1	Nat Commun 12, 3502, 2021
Pt@ A&R-TiO_2	5.4	Commun Mater 6, 15, 2025
Pt_1/WC_x@CNTs	4.2	Nano Lett. 25, 3066–3074, 2025
Pt_1/OLC	≈7.5	Nat Energy 4, 512–518, 2019
$PdPt_{2L}$	3.64	Small, 20, 2406935, 2024
Pt (110)-Ni_3N	≈1.05	Nano Res. 16, 174–180, 2023
Pt/MoB-M	10.06	J Colloid Interf Sci. 678, 2025
Pt/CNT45	18.76	Small, 2411181, 2025
$Au_{38.4}$@$Au_{4.1}Pt_{57.5}$	63.8	Nanoscale,15, 4378, 2023
Au_1Pt_4 Cl	13.8	J. Mater. Chem. A, 13, 7721, 2025
Pt_3Fe/NMCS-A	1.94	Adv Mater. 35(41):e2303030, 2023
PtAuCu NWs	≈6.5	Nano Res, 16(8): 10742, 2023

Table S3. Electrochemical impedance spectroscopy (EIS) parameters obtained by fitting to a Randles circuit model for AuPt nanowires with different Pt loadings under dark and 525 nm LED-illuminated conditions. In all cases, the charge-transfer resistance (Rct) decreases under illumination, indicating enhanced interfacial charge transport upon plasmon excitation. The effect is most pronounced for Au98.5Pt1.5, consistent with its superior light enhanced HER performance.

**Sample / light condition**		**Rs (Ω cm^2)**	**Rp (Ω cm^2)**	**CPE 1**		**Rct (Ω cm^2)**	**CPE 2**	
				Q	n		Q	n
**$Au_{99}Pt_1$**	Dark	0.91	0.16	5.7E-7	0.92	4.02	2E-4	0.81
	525 nm	0.92	0.17	5.4E-7	0.95	3.52	1.9E-4	0.81
**$Au_{98.5}Pt_{1.5}$**	Dark	1.10	0.58	1.0E-3	0.60	0.74	0.026	0.70
	525 nm	1.22	0.55	2.3E-3	0.40	0.53	0.013	0.81
**$Au_{92}Pt_8$**	Dark	1.21	0.74	3.0E-4	0.70	0.99	8.9E-3	0.66
	525 nm	1.20	0.38	4.5E-4	0.71	0.54	7.8E-3	0.94
